# Transcription factor switching drives subtype-specific pancreatic cancer

**DOI:** 10.1038/s41588-025-02389-7

**Published:** 2025-10-30

**Authors:** Shalini V. Rao, Lisa Young, Danya Cheeseman, Sean Flynn, Niklas Krebs, Dominique-Laurent Couturier, Stephanie Mack, Rebecca Brais, Jill Temple, Amy Smith, Evangelia Papachristou, Catarina Pelicano, Chandra Sekhar Reddy Chilamakuri, Krzysztof Herka, Hideo Baba, Luay Farah, Phyllis F. Cheung, Jens Siveke, Stéphane Guerrier, Luca Insolia, Michael Gill, Emily Archer Goode, Steven Kupczak, Yi Cheng, Giacomo Borsari, Duncan Jodrell, Clive D’Santos, Alasdair Russell, Barbara T. Grünwald, Eva Serrao, Igor Chernukhin, Jason S. Carroll

**Affiliations:** 1 Cancer Research UK Cambridge Institute, University of Cambridge, Cambridge, UK; 2 Department of Clinical and Biomedical Sciences, Faculty of Health and Life Sciences, University of Exeter, Exeter, UK; 3 West German Cancer Center, Experimental Urology, University Hospital Essen, Essen, Germany; 4 Medical Research Council Biostatistics Unit, University of Cambridge, Cambridge, UK; 5 Cambridge University Hospitals NHS Foundation Trust, Cambridge, UK; 6 Department of Pathology, University Hospital Essen, Essen, Germany; 7 Bridge Institute of Experimental Tumor Therapy (BIT) and Division of Solid Tumour Translational Oncology, German Cancer Consortium (DKTK), University Hospital Essen, Essen, Germany; 8 German Cancer Consortium (DKTK) partner site Essen, a partnership between German Cancer Research Center (DKFZ) and University Hospital Essen, Essen, Germany; 9 Faculty of Science, University of Geneva, Geneva, Switzerland; 10 Addenbrooke’s hospital, Cambridge, UK

**Keywords:** Cancer, Cancer

## Abstract

Emerging evidence suggests that lineage-specifying transcription factors control the progression of pancreatic ductal adenocarcinoma (PDAC). We have discovered a transcription factor switching mechanism involving the poorly characterized orphan nuclear receptor HNF4G and the putative pioneer factor FOXA1, which drives PDAC progression. Using our unbiased protein interactome discovery approach, we identified HNF4A and HNF4G as reproducible, FOXA1-associated proteins, in both preclinical models and Whipple surgical samples. In the primary tumor context, we consistently find that the dominant transcription factor is HNF4G, where it functions as the driver. A molecular switch occurs in advanced disease, whereby HNF4G expression or activity decreases, unmasking FOXA1’s transcriptional potential. Derepressed FOXA1 drives late-stage disease by orchestrating metastasis-specific enhancer–promoter loops to regulate the expression of metastatic genes. Overall survival is influenced by HNF4G and FOXA1 activity in primary tumor growth and in metastasis, respectively. We suggest that the existence of stage-dependent transcription factor activity, triggered by molecular compartmentalization, mediates the progression of PDAC.

## Abstract (web summary)

Pancreatic cancer progression is driven by a switch from HNF4G-driven transcriptional activity in primary disease to FOXA1-mediated transcription in the metastatic setting.

## Main

Despite recent advances in the treatment of pancreatic ductal adenocarcinoma (PDAC), median survival remains less than 12 months^1^. Recent large-scale genomic approaches have shown that PDAC is composed of at least two distinct molecular subtypes, based on transcriptomic signatures, and these are termed the classical and squamous (or basal/mesenchymal) subtypes. Although it is well described that PDAC harbors gene mutations (that is, KRAS G12D and p53 R172H) that initiate the disease, little is known about the influence of lineage-defining transcription factors on the PDAC molecular subtypes^1–5^.

Pioneer factors (PFs) are a specialized type of transcription factors that can bind directly to condensed chromatin and create enhancer elements within the genome, and changes in the expression levels or fidelity of these specialized transcription factor TF can alter cell lineage. The archetypal PF is the forkhead transcription factor FOXA1 (refs. ^6–9^). In hormone-dependent breast cancer, FOXA1 functions in a physical complex with GATA3 and the nuclear receptor (NR) estrogen receptor (ER), whereas in prostate cancer, FOXA1 forms a transcriptional complex with GATA2 and the NR androgen receptor (AR)^9^. Seminal work in metastatic organoid models (mouse) of PDAC demonstrated that elevated FOXA1 and GATA5 led to the creation of new enhancer elements, which were associated with increased metastatic potential^10^.

The HNF4 families of transcription factors have been implicated in an endodermal identity^2^ and in the development of PDAC, with both HNF4A and HNF4G being differentially expressed in the classical subtype of the disease. At a minimum, these two transcription factors could function as biomarkers of this common subtype of PDAC, but as developmental transcription factors^11^, they potentially could have a functional role in tumor progression. Supporting this, HNF4A was previously shown to cooperate with GATA6 (another transcription factor commonly amplified in PDAC) to maintain a metabolic signature of the classical subtype^12^. Very little is known about HNF4G, although it has been shown to impact cellular proliferation and invasion^13–16^. The SMAD4 deficiency in PDAC has been shown to increase HNF4G expression and oncogenic potential^15,17^. One of the most statistically enriched germ-line variants is at the HNF4G gene locus and is linked with increased HNF4G expression and increased risk of developing PDAC^18^.

We sought to leverage the link between FOXA1 and PDAC metastasis to purify FOXA1-associated proteins, thereby definitively identifying and characterizing the protein complex involved in disease progression. We were specifically interested in discovering NR transcription factors in PDAC, similar to what is observed between FOXA1 and ESR1 in breast cancer and between FOXA1 and AR in prostate cancer. We identify HNF4 family members as consistent FOXA1-associated proteins and show a stage-specific mutually exclusive role for HNF4G and subsequently for FOXA1 in distinct stages of the disease, from primary tumor to metastasis.

## Results

### Mapping enhancer regions in normal adjacent tissue versus PDAC

**Fig. 1:**
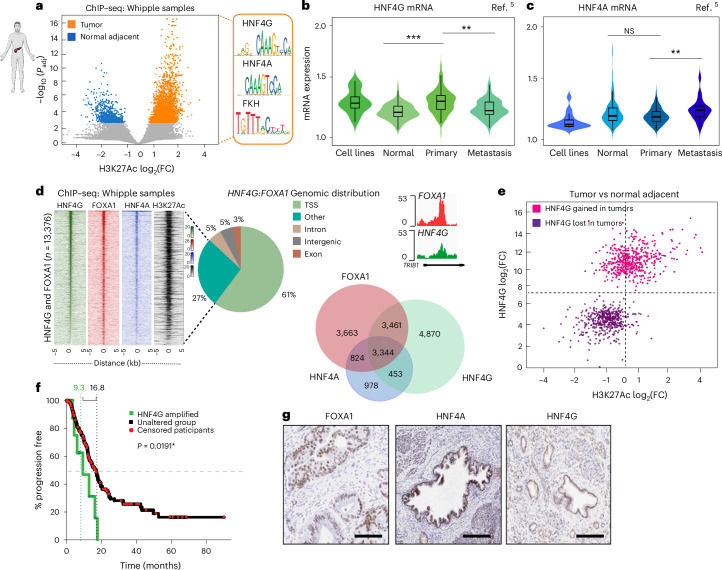
Discovery of the HNF4A and HNF4G orphan NRs. **a**, ChIP–seq was performed in patient samples taken from Whipple surgery of normal adjacent (*n* = 4) and PDAC samples (*n* = 6). Volcano plot represents enriched sites of H3K27Ac, with normalized intensities of called peaks (3,000 sites) in normal and tumor samples. The data have been analyzed using negative binomial GLM as a part of the DiffBind package. The color code shows the significant thresholds log_10_(*P*_adj_). Panel **a** was created with BioRender.com. **b**,**c**, Box plots show the gene expression levels of HNF4G and adapted from ref. ^5^. Two-sided Kruskal–Wallis test was performed. HNF4G expression primary and normal tissues (*P*_adj_ < 1.4 × 10^−14^), primary and metastases (*P*_adj_ < 1.4 × 10^−5^). **P ≤ 0.03, ***P ≤ 0.01. Normal tissue = 134, primary = 145, metastasis = 61. Metadata for the plots, median—0.851, 0.633, 0.898, 0.673; 25th percentiles—0.734, 0.530, 0.700, 0.582; 75th percentiles—1.003, 0.781, 1.035, 0.879; lower whisker—0.404–0.734, 0.223–0.530, 0.377–0.700, 0.259–0.582; upper whisker—1.003–1.388, 0.781–1.149, 1.035–1.524, 0.879–1.295; upper adjacent value—1.388, 1.149, 1.524, 1.295; lower adjacent value—0.404, 0.223, 0.377, 0.259; box bounds—0.734–1.003, 0.530–0.781, 0.700–1.035, 0.582–0.87. **d**, Heatmaps show coverage intensities mapped onto overlapped sites between FOXA1, H3K27Ac, HNF4A and HNF4G. An illustrative snapshot of a genomic region showing common FOXA1 and HNF4G binding sites. Venn diagram showing the overlapping sites between FOXA1, HNF4G and HNF4A peaks across four independent primary tumors. Distribution of genomic features across FOXA1 and HNF4G binding sites in primary tumors (*n* = 4). IGV screenshots representing FOXA1 and HNF4G binding from a patient sample. **e**, ChIP–seq was performed in patient samples of normal adjacent (*n* = 4) and PDAC samples (*n* = 6) to pull down HNF4G and H3K27Ac. Correlation of log_2_-fold changes between H3K27Ac and HNF4G ChIP–seq intensities (tumor versus normal adjacent). **f**, Kaplan–Meier plot and log-rank test were used to assess the survival curves for patients with HNF4G amplification versus those that were copy number neutral from TCGA data. **g**, IHC staining performed on frozen sections (*n* = 10). Representative images of FOXA1, HNF4G and HNF4A restricted to the epithelial cell compartment from one patient are presented. GLM, generalized linear model; FC, fold change.

**Extended Data Fig. 1:**
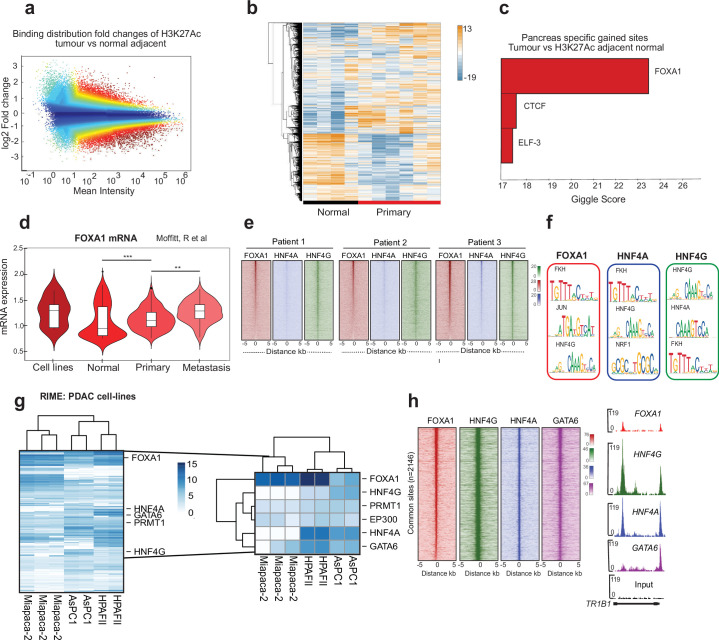
Discovery of FOXA1-associated orphan nuclear receptors. **a**. The MA plot shows differential binding intensity in H3K27Ac ChIP-seq between tumor and normal adjacent tissue. Scatter plot highlights the top enriched TF binding in the conditions. **b**. ChIP-seq was performed in Whipple samples of normal adjacent (n = 4) and PDAC samples (n = 6). Heatmap represents hierarchical clustering of H3K27Ac-normalized intensities of called peaks (3,000 sites) in normal and tumor samples. **c**. Giggle cistrome analysis reveals FOXA1 as a dominant TF in the H3K27Ac regions abundant in tumor tissue. Higher Giggle scores indicate more enrichment. **d**. Box plots show the gene expression levels of FOXA1 in clinical samples. Data adapted from ref. ^5^. **e**. Tagged intensity heatmaps showing binding of FOXA1, HNF4G and HNF4A across patient samples. **f**. Top enriched motifs discovered during analysis of called peaks from clinical samples (n = 4). **g**. RIME in a panel of pancreatic cancer cell lines. Three biological replicates of the FOXA1 RIME and one pooled immunoglobulin G (IgG) control RIME were included in each experiment. To identify specific interactors, a label-free quantification method was used. Significantly enriched interactors are highlighted on the heatmap padj < 0.02. **h**. Heatmaps illustrate normalized coverage density at common ChIP-seq sites for FOXA1, HNF4G, HNF4A and GATA6 in the AsPC-1 cell line model. UCSC tracks show common binding sites for the respective pull downs.

We adopted an unbiased approach to identify driving transcription factors in PDAC and performed chromatin immunoprecipitation sequencing (ChIP–seq) of the active enhancer mark H3K27Ac on surplus tissue obtained after the resection of primary PDAC (Whipple surgical samples). We identified all genomic intervals with substantial fold changes in H3K27Ac signal between PDAC tumors (*n* = 6) and normal adjacent tissue (*n* = 4; Fig. 1a). A median of 65,010 H3K27Ac peaks per sample (range = 43,909–76,827) was observed. Enriched enhancer regions, specifically in primary PDAC lesions, are presented in Fig. 1a, and we termed these sites as PDAC-enriched candidate regulatory elements (PDAC-CREs). Unsupervised hierarchical clustering revealed a substantial H3K27Ac divergence between tumors and the adjacent normal tissue (Fig. 1a and Extended Data Fig. 1a,b). Differential transcription factor binding at the tumor-specific regions was assessed using the cistrome DB toolkit, an approach that identifies binding site similarity with known experimental transcription factor datasets (Extended Data Fig. 1c), with FOXA1 being the top correlated transcription factor from published ChIP–seq datasets. Analysis of the H3K27Ac sites showed that the most enriched motifs within all experimentally mapped enhancer elements from patient samples were hepatocyte nuclear factor (HNF) motifs (HNF4G and HNF4A) and Forkhead (that is, FOXA1; Fig. 1a), confirming that these two classes of transcription factors constitute the lineage-defining factors in these patient tumor samples. The analysis of clinical datasets revealed that HNF4A levels did not differ between normal and primary tumor samples but were modestly increased in metastatic samples. In contrast, we observed elevated HNF4G levels in primary tumors compared to the normal adjacent tissue, but surprisingly HNF4G mRNA levels in metastases were closer to those observed in normal tissue (Fig. 1b,c)^5^. In line with the previous reports, FOXA1 levels were found to be elevated in both the metastatic and the tumor cohorts (Extended Data Fig. 1d)^10^. We conducted ChIP–seq of HNF4G, HNF4A and FOXA1 from Whipple surgical samples from PDAC patients (*n* = 6; Fig.1d and Extended Data Fig. 1e,f), revealing 3,344 reproducible, consensus binding sites where all three transcription factors cobound at the same genomic regions (Fig. 1d and Extended Data Fig. 1e–f). These cobound regions were enriched at promoter proximal regions of the genome (Fig. 1d). We also observed a total of 3,461 regions where FOXA1 and HNF4G were corecruited, without any HNF4A binding. The top enriched motifs for the respective pull downs were HNF4 and FOXA1 (Extended Data Fig. 1f), suggesting that these transcription factors bind directly to DNA using consensus motifs.

The transcription factor mapping data suggested that the regions of greatest enhancer activity (as defined by the H3K27Ac data) were preferentially occupied by FOXA1 and HNF4G. A comparison of HNF4G ChIP–seq from normal adjacent tissue (Fig. 1e) showed that tumor-specific enhancers were occupied by HNF4G. In support of a role for HNF4G, the genomic region encoding the HNF4G gene was shown to be amplified in 9% PDAC patients (Fig. 1f). Immunohistochemistry (IHC) analysis of FOXA1, HNF4A and HNF4G from primary PDAC tissue samples confirmed coexpression of these transcription factors, only in the cancer epithelial cells (Fig. 1g). These findings suggest that HNF4G mRNA levels and transcriptional activity increase when cells transition from normal to cancer, that HNF4G and FOXA1 are the key lineage-defining transcription factors in PDAC and that amplification of HNF4G is a common event that correlates with poor outcome.

### Discovery of NRs in the FOXA1 interactome

**Fig. 2:**
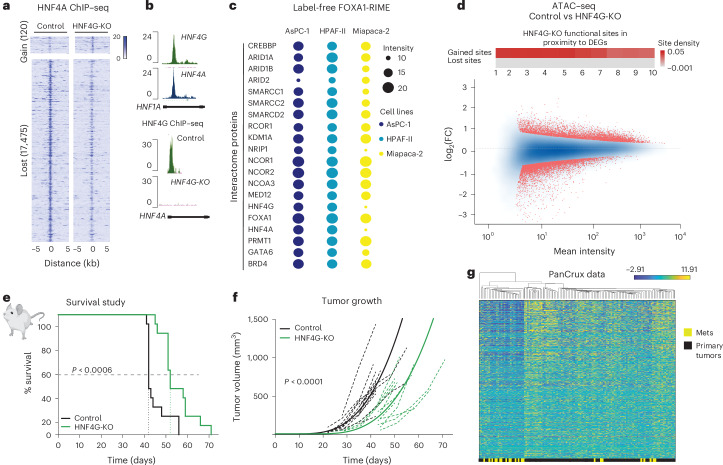
Validation of HNF4G as a therapeutic target. **a**, HNF4A ChIP–seq performed in HPAF-II human tumor cell line +/− HNF4G-KO. Heatmaps illustrate normalized coverage density at lost and gained ChIP–seq sites revealed with DiffBind statistical test at *P*_adj _< 0.05. **b**, UCSC genome browser tracks representing binding of HNF1A and HNF4A in the control and HNF4G-KO HPAF-II cells (chr20: 44,282,766–44,452,506). **c**, RIME in a panel of pancreatic cancer cell lines. Three biological replicates of FOXA1-RIME and one pooled IgG control RIME were included in each experiment. To identify specific interactors, a label-free quantification method was used. Substantially enriched interactors are highlighted on the heatmap *P*_adj_ < 0.02. Panel **c** was created with BioRender.com. **d**, MA plot illustrates the changes in ATAC–seq open chromatin regions in HNF4G-KO versus control (Cas9) HPAF-II cells. Data combine four independent biological replicates. Statistically significant values are highlighted in red. The heatmap bar above shows correlation in the vicinity between gained ATAC events and active gene loci in patient samples (PanCuRX dataset)^37^. **e**,**f**, HPAF-II cell was orthotopically injected into the pancreas of NSG mice. Data from survival (**e**) and tumor growth (**f**) analyses of ultrasound images of tumors are represented for the control (Cas9) and HNF4G-KO tumors. *P* value of <0.0006 calculation was generated from a two-sided two-sample Mann–Whitney–Wilcoxon test to assess if the median of the difference between the samples of each group is different from 0. *P* values were calculated with a Welch test with multiplicity corrections for the growth curves were assessed by a likelihood ratio test *P* value of <0.0001. The lighter lines indicate mouse-specific longitudinal tumor volume measures, color-coded by specific groups. **g**, DEGs derived from the HNF4G-KO RNA-seq analysis were evaluated by integrating clinical data from the PanCuRX study. Downregulated genes from in Deseq2 test of HNF4G-KO versus control samples were used to visualize expression patterns in the classical subtype tumors from the PanCuRX dataset. Hierarchical clustering was used to order clinical samples column-wise, while the gene expression order was preserved according to descending expression values of RNA-seq. The color bar at the bottom depicts the separation pattern between classical primary tumors and metastatic samples (*n* = 138). IgG, immunoglobulin G; DEGs, differentially expressed genes.

**Extended Data Fig. 2:**
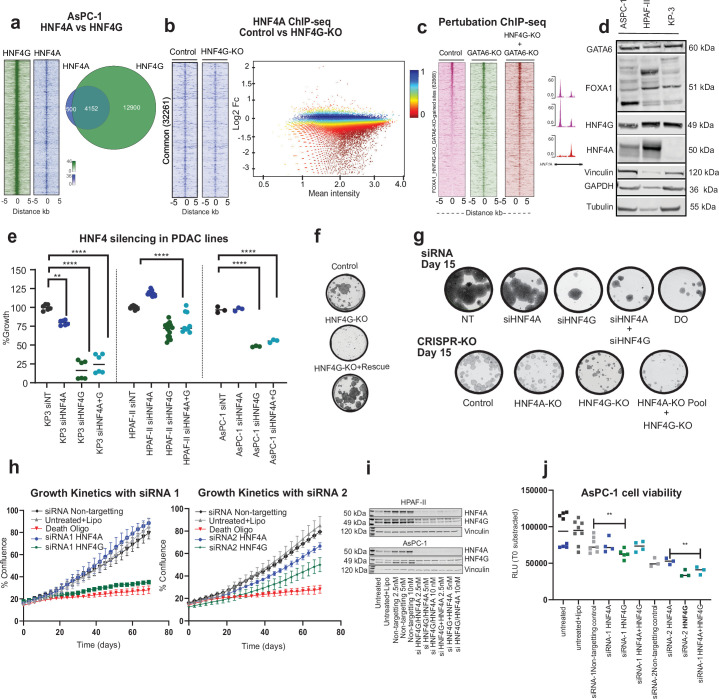
Functional investigation of HNF4A and HNF4G as therapeutic targets. **a**. Heatmaps illustrate normalized coverage density at common ChIP-seq sites for HNF4G, HNF4A in the AsPC-1 model. Venn diagram shows shared binding between the factors. **b**. HNF4A ChIP-seq performed in HPAF-II human tumor cell line +/− HNF4G-KO. Heatmaps illustrate normalized coverage density at common ChIP-seq sites. HNF4A binding sites that were either common, gained or lost in the +/− HNF4G-KO context represented as an MS plot (on the right), subsets measured occupancy for each context using DiffBind at padj < 0.05 threshold. **c**. Perturbation ChIP-seq performed in HPAF-II human tumor cell line with the following conditions control (pink-GATA6 I.P), GATA-KO (green-HNF4G-KO I.P), HNF4G-KO + GATA6-KO (red-FOXA1 I.P). **d**. Western blots in PDAC cell lines showing expression levels of FOXA1, GATA6, HNF4A and HNF4G. Normalization controls include vinculin, GAPDH and tubulin. **e**. siRNA experiments performed in the KP-3, HPAF-II and AsPC-1 cells transfected with 10 nM siRNA for 72 h using stealth siRNA. Cell viability was assessed using CellTiter-Glo. Data presented are from three independent biological replicates. Significance was calculated with multiple comparison ANOVA (p-value ****<0.01, **<0.02). **f**. Colony formation assay performed in CRISPR clones/pools targeting HNF4G (CRISPR clones), HNF4G-rescue in the HPAF-II cells. **g**. Colony formation assay (10 nM siRNA and CRISPR KO in HPAF-II cells) was performed in tandem with growth experiments. Cells were monitored, stained with crystal violet at endpoint (day 15). Representative images in HPAF-II cells are from an independent well of a 24-well plate. **h**. An independent set of siRNAs 1 (smart pool), siRNAs 2 (stealth pool) were used to treat HPAF-II cell line models, at 10 nM to perform the proliferation assays using the Incucyte (72 h). Non-targeting (NT), death oligo (DO) HNF4G and HNF4A siRNAs were used. **i**. Representative western blots showing the knockdown efficiency using the stealth siRNA targeted towards HNF4A and HNF4G in the HPAF-II cells at endpoint (72 h) compared to untreated cells and non-targeting control (NT). **j**. An independent set of 2 siRNAs (smart pool and stealth siRNA) were used in the AsPC-1 cell line, at 10 nM. Non-targeting (NT), HNF4G and HNF4A siRNAs were used, and CellTiter-Glow was performed at the endpoint (72 h). Source data

HNF4G and HNF4A are members of the NR superfamily of transcription factors, which is the only readily druggable class of transcription factors due to the presence of a ligand-binding domain. Although our initial discoveries from patient samples suggested that HNF4A and HNF4G could co-occupy many genomic regulation elements, we could show that HNF4A binding is dependent on HNF4G (Fig. 2a,b and Extended Data Fig. 2b), with global HNF4A binding sites decreased when HNF4G was silenced. We observed that HNF4G binds directly to CREs at the HNF4A genomic locus, implying direct regulation of HNF4A expression (Fig. 2b). Therefore, although HNF4A represents one of the best biomarkers of the classical subtype of PDAC, our data suggest that HNF4A activity is functionally downstream of HNF4G.

**Extended Data Fig. 3:**
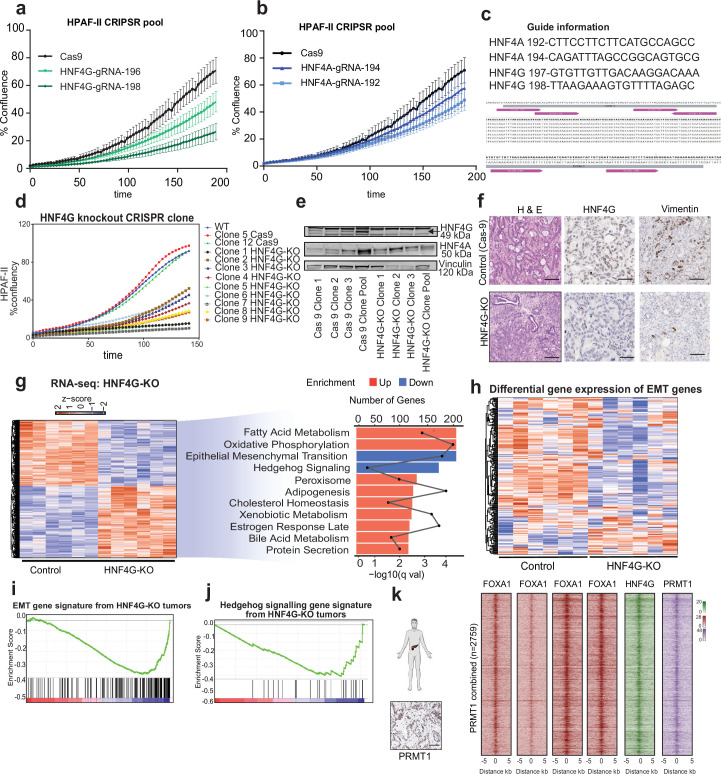
HNF4G-KO results in significant changes in the transcriptome analysis. **a**&**b** Two independent CRISPR guides were used towards the KO of HNF4G and HNF4A in HPAF-II cell line models, proliferation assays were performed using the Incucyte (72 h). **c**. Guide information and snap gene information for the guides used (HNF4A and HNF4G). **d**. Proliferation assay performed in 9 independent CRISPR HNF4G-KO clones compared to control (Cas9) and wildtype (WT) cells using the Incucyte. **e**. Western blots performed in the HPAF-II cells to determine knockout efficiency of the gRNAs targeting HNF4A and HNF4G. The western blots were normalized using vinculin as a loading control. **f**. IHC staining of the control and HNF4G-KO tumors stained for H&E, HNF4G and vimentin. A representative image of the staining is shown. **g**. Results of mRNA expression analysis comparing control versus HNF4G-KO tumors. Heatmap highlights the 4,247 most differentially regulated genes associated with padj value < 0.01, evaluated with DESeq2. GSEA analysis of mRNA expression revealed meaningful matches with gene sets from the MySigDB hallmark database. **h**. Differential pathway analysis of the HNF4G-KO tumors, highlighted in red—upregulated, blue—downregulated GSEA gene expression sets. Heatmap represents differentially regulated genes from the EMT pathway extracted from gene set enrichment analysis (GSEA). **i**&**j** GSEA analysis comparing HNF4G-KO tumors with control. Snapshots of the top two downregulated pathways. **k**. Heatmaps anchored on PRMT1 (n = 2759 sites) showing co-binding of FOXA1 and HNF4G at these sites in multiple patient tumor samples. Representative image of PRMT1 IHC staining in patient samples used for ChIP-seq. Panel **k** was created with BioRender.com. Source data

Our data suggest that HNF4G was essential for the growth of classical PDAC models, whereas silencing of HNF4A alone was not sufficient to affect cell proliferation (Extended Data Figs. 2e–h and 3a–e). We subsequently used rapid immunoprecipitation and mass spectrometry of endogenous (RIME) protein complexes, our method for unbiased discovery of endogenous protein complexes^19–21^ and purified FOXA1 as the ‘bait’ to discover associated protein complexes (Fig. 2c). This was applied to two classical (HPAF-II and AsPC-1) PDAC cell line models (where HNF4A and HNF4G are highly expressed) and one mixed subtype model as a control (Miapaca-2; Fig. 2c, Extended Data Fig. 1g and Supplementary Table 1). After FOXA1-RIME, we identified numerous subunits of the ATP-dependent SWI/SNF complex, known NR cofactors, including CBP, NCOA3 and NCOR1/NCOR2, as well as GATA6 (ref. ^21^). Notably, this suggests that FOXA1 is present in both major molecular subtypes of PDAC and many of the FOXA1-associated coregulatory proteins were the same, except for HNF4A and HNF4G, which were only seen as FOXA1-associated proteins in models of the classical subtype of the disease. We validated this observation in additional models (Supplementary Table 1)^2,22^. To confirm that HNF4A and HNF4G were in a complex with both of the metastasis-associated transcription factors, FOXA1 and GATA6 (ref. ^23^), we also conducted RIME of GATA6 in the AsPC-1 classical model, which confirmed unbiased discovery of interactions among HNF4A, HNF4G, GATA6 and FOXA1 (Supplementary Table 1). The genomic binding profiles and physical interactions among HNF4G, FOXA1, HNF4A and GATA6 were validated in additional models of the classical subtype (Extended Data Figs. 1h and 2a).

We hypothesized that FOXA1 was the PF for HNF4G (and possibly HNF4A), as seen with other NRs in breast and prostate cancer that require FOXA1. However, our in vitro data suggest that, although HNF4G was consistently required for viability of classical PDAC cell line models (Extended Data Figs. 2e–j and 3a,b), FOXA1 was not required. These data imply that FOXA1 might not be the PF for HNF4G, because depletion of FOXA1 should phenocopy HNF4G depletion. We also explored GATA6 as a potential PF for HNF4G, but silencing of GATA6 did not result in decreased HNF4G binding sites (Extended Data Fig. 2c) and instead depletion of GATA6 resulted in substantial gains in HNF4G binding sites, suggesting that GATA6 could be a negative regulator of HNF4G activity. We also found little evidence that HNF4A was required for the growth of classical models (Extended Data Fig. 2e–j). We, therefore, focused on HNF4G as a chromatin regulatory protein and specifically silenced HNF4G and subsequently conducted the assay for transposase accessible chromatin with high-throughput sequencing (ATAC–seq; Fig. 2d). We found 5,203 regions that were closed and 4,275 regions that were opened after HNF4G depletion. We could also confirm that the HNF4G-dependent chromatin accessibility regions were adjacent to the HNF4G-dependent target genes (Fig. 2d), suggesting that HNF4G is responsible for creating the enhancers that drive the target gene expression events. Therefore, HNF4G is required for maintaining chromatin accessibility and HNF4G activity is not dependent on FOXA1 or on GATA6.

### HNF4G mediates primary tumor growth but suppresses metastatic genes

We sought to explore the role of HNF4G in more complex tumor models of human disease. CRISPR knockout (KO) clones were generated in the HPAF-II cells (Extended Data Fig. 3d). We orthotopically injected HNF4G-KO pooled clones or control cells into the pancreas of NOD SCID-γ (NSG) mice and observed a significant increase (*P* < 0.0006) in the median survival of mice bearing HNF4G-KO tumors compared to control tumors (Fig. 2e). The HNF4G-KO resulted in slower tumor growth compared to control (Cas9) tumors (*P* < 0.0001) and substantially smaller tumors (Fig. 2f), confirming that HNF4G is required for optimal tumor growth. We conducted RNA-seq on the orthotopic tumor samples (Extended Data Fig. 3f–j) and identified an HNF4G-specific target gene signature that was assessed in the PanCuRx clinical cohort^24^. Using unsupervised hierarchical clustering, the HNF4G target genes (discovered in our orthotopic tumor experiments; Fig. 2g) were specifically expressed in primary tumors, as opposed to metastatic samples (Fig. 2g), implying that the HNF4G target genes were restricted to primary tumor contexts. The HNF4G target genes that were differentially expressed in the PanCuRx primary tumor samples were adjacent to the genomic regions that were ‘opened’ by HNF4G in our cell line models (Fig. 2d, red panel above MA plot), confirming that HNF4G mediates chromatin accessibility at gene targets that are commonly expressed in patient samples.

The findings show that FOXA1 cobinds with HNF4G in the classical subtype of PDAC, but that FOXA1 is not a PF in this PDAC subtype and, instead, the key transcription factor driving primary tumor growth appears to be HNF4G. Thus, HNF4G can induce chromatin opening at the target genes that are consistently expressed in patient primary tumor samples, but these genes are decreased in expression in metastases, mimicking the expression profile of HNF4G mRNA (Fig. 1b).

### PRMT1 is a substantial, functional interactor of HNF4G

**Fig. 3:**
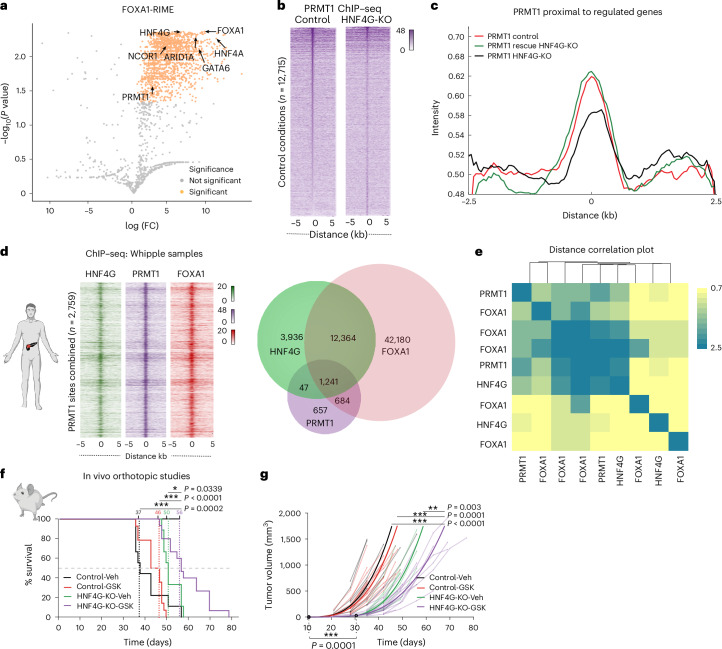
PRMT1 physically associates with the FOXA1 and HNF4G interactome. **a**, Volcano plot represents FOXA1-RIME candidates discovered in HPAF-II cells. The average of identified peptides in three biological replicates was calculated and proteins absent in IgG control samples were filtered for <3 unique peptides, *P* values of 0.003. Proteins of interest are highlighted. **b**, Signal intensity plots generated from PRMT1 ChIP–seq in control and HNF4G-KO binding regions. **c**, Intensities of PRMT1 binding coverage measured at open sites (H3K27Ac) in proximity to HNF4G-regulated genes found in control, HNF4G-KO, HNF4G-KO + rescue conditions. **d**, ChIP–seq tag density heatmap of FOXA1, HNF4G and PRMT1 co-occupancy at PRMT1 binding sites from a representative patient tumor sample. Venn diagram is an overlap of binding sites of these factors from one representative patient. Panel **d** was created with BioRender.com. **e**, Distance correlation heatmap of ChIP–seq locations illustrates cobinding similarity of PRMT1, FOXA1 and HNF4G from three independent Whipple surgical samples. **f**,**g**, Control and HNF4G-KO cells were orthotopically implanted into the pancreas of mice. Mice with palpable tumors were then randomized into four groups and treated with GSK3368715 (oral administration of 75 mg kg^−1^ five doses per week) for 4 weeks, with *n* = 9 mice for control vehicle and HNF4G-KO vehicle arms and for control + GSK3368715 and HNF4G-KO + GSK3368715 arms *n* = 12. *P = 0.0339, ***P < 0.0001 and ***P = 0.0002 were calculated with a Welch test with multiplicity corrections (**f**). **P = 0.003, ***P = 0.0001 and ***P < 0.0001 of random intercept piecewise model parameters were defined by means of Wald *t* tests (**g**). The lighter lines indicate mouse-specific longitudinal tumor volume measures, color-coded by specific groups.

HNF4G is a poorly studied NR, and it is currently classified as an ‘orphan’ NR, because no endogenous ligands have been implicated for these orphan receptors^25,26^. Because there are no known or validated chemical inhibitors of HNF4G, we re-examined our quantitative RIME (Fig. 3a) data, with the goal of finding potential druggable enzymes that are associated with the FOXA1, GATA6 and HNF4G protein complex. We found protein arginine methyltransferase (PRMT1) to be a substantial and reproducible interactor of FOXA1 and HNF4G (Fig. 3a). Previous data had implicated PRMT1 as an essential gene in a subset of PDAC patient-derived xenograft tumors^27^. The transcriptionally active HNF4G-specific enhancer elements were cobound by PRMT1, as measured by ChIP–seq, and PRMT1 binding was dependent on HNF4G (Fig. 3b,c). We could also show PRMT1 cobinding with HNF4G (and FOXA1) in the Whipple surgical samples (*n* = 3; Fig. 3d,e and Extended Data Fig. 3k).

**Extended Data Fig. 4:**
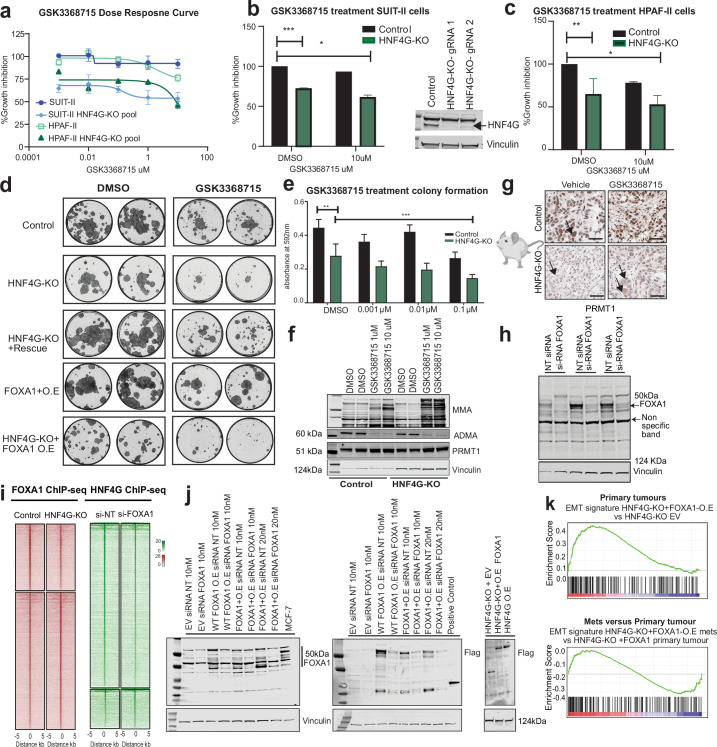
Functional validation of PRMT1 & HNF4G dependency. **a**. Dose–response curves for GSK3368715 treatment in the SUIT-II (+/− HNF4G-KO) and HPAF-II cells (+/− HNF4G-KO) performed at 72 h using the CellTiter-Glow (CTG). **b**. Treatment of SUIT-II (+/− HNF4G-KO) cells with GSK3368715 (10 µM) for 72 h endpoint CTG and western blot representing CRISPR KO efficiency of HNF4G in SUIT-II cells P-values *** <0.02, * <0.05. **c**. Treatment of HPAF-II (+/− HNF4G-KO) cells with GSK3368715 (10 µM) for 72 h endpoint CTG. **d**. Colony formation assay (day 15) performed in the HPAF-II cells with control, HNF4G-KO, HNF4G-O.E, FOXA1-O.E and HNF4G-KO + FOXA1 (n = 3) cell were stained with crystal violet. **e**. Crystal violet-stained colonies were solubilized and absorbance was measured (592 nm) for the control and HNF4G-KO conditions treated with GSK3368715. Two-way ANOVA analysis was performed on n = 3 independent experiments padj <0.02. **f**. Western blots were performed in the HPAF-II cells treated with +/− GSK3368715 for 72 h. Blots represent monomethyl-arginine (MMA), asymmetric dimethyl-arginine antibody (ADMA) and PRMT1 protein levels. Western blots are normalized to vinculin. **g**. Representative image of PRMT1 IHC staining in control, HNF4G-KO tumors +/− GSK3368715 treatment. **h**. Western blots were performed in the HPAF-II cells treated with non-targeting siRNA and FOXA1-siRNA cells in the HPAF-II (treated for 72 h at 20 nM). Blots represent FOXA1 protein expression levels from three independent biological replicates. **i**. Signal intensity heatmaps of FOXA1 binding in the control and HNF4G-KO HPAF-II cells. Signal intensity heatmaps of HNF4G binding in the non-targeting (NT) siRNA and FOXA1-siRNA-transfected HPAF-II cells (transfected for 72 h at 20 nM). Data presented are the cumulative intensity from three independent biological replicates. **j**. Western blots were performed in the HPAF-II cells with FOXA1 and HNF4G overexpression (FOXA1-O.E & HNF4G-O.E) and FOXA1-siRNA cells (transfected for 72 h with 10 nM and 20 nM siRNA). Blots represent Flag, FOXA1, HNF4G protein expression levels from one representative biological replicate. **k**. GSEA analysis was performed on tumors harvested from HPAF-II orthotopic tumors, comparing HNF4G-KO + FOXA1-O.E primary tumors versus HNF4G-KO-EV primary tumors. HNF4G-KO + FOXA1-O.E metastasis (mets) versus HNF4G-KO + FOXA1-O.E primary tumors. GSEA snapshot representing EMT gene signature is presented. Source data

PRMT1 has been previously shown to be essential for the viability of specific PDAC patient-derived xenograft tumor models^27^. We could show that the same Type I-PRMT inhibitor (GSK3368715) was more effective at inhibiting growth in the HNF4G-KO models (Extended Data Fig. 4a–c), which was associated with an accumulation of the monomethylation mark, the basal state when type I PRMTs are inactive (Extended Data Fig. 4d–f). FOXA1 overexpression (OE) did not appreciably alter sensitivity to the PRMT inhibitor. We conducted a four-arm in vivo efficacy study in which control or HNF4G-KO cells were injected orthotopically into the pancreas of NSG mice and subsequently treated with vehicle or 75 mg kg^−1^ GSK3368715 (Fig. 3f,g). We observed a substantial survival advantage of 19 days in HNF4G-KO tumor-bearing mice (*P* < 0.03) that were treated with GSK3368715 (Fig. 3f). These data confirm a functional dependence between the NR HNF4G and the methyltransferase PRMT1 that can potentially be exploited therapeutically.

### HNF4G is the dominant transcription factor in primary tumors

Our data so far suggest that HNF4G can cobind with FOXA1 to the same genomic regions, but only HNF4G is essential for tumor growth, in part, by recruitment of PRMT1. To explore the potential connection further, we depleted FOXA1 and mapped HNF4G binding sites, or vice versa (Extended Data Fig. 4i). There was no appreciable loss of FOXA1 binding when the NR (HNF4G) was depleted (Extended Data Fig. 4i) and, notably, we did not see any reproducible decrease in HNF4G binding when FOXA1 was silenced (Extended Data Fig. 4i), confirming the evolving conclusion that FOXA1 is not a PF for the lineage-defining NR (that is, HNF4G) in primary PDAC.

**Fig. 4:**
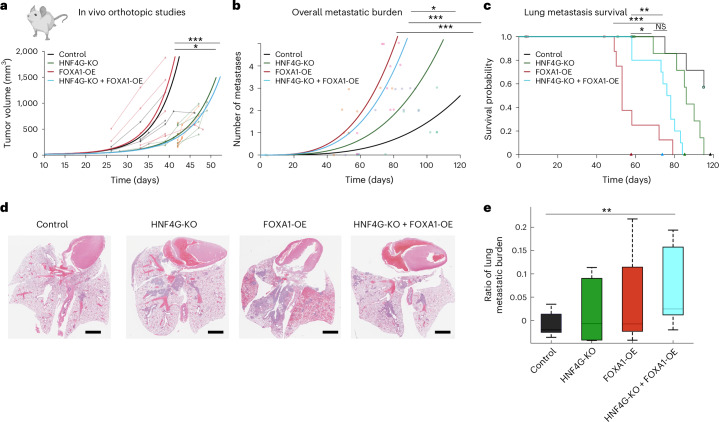
Transcriptional switch from HNF4G-dependent primary tumor to FOXA1-induced metastatic progression. **a**, Cells were orthotopically implanted into the pancreas of NSG mice (*n* = 5) for the following conditions: control, HNF4G-KO, FOXA1-OE and HNF4G-KO + FOXA1. Multiplicity *P*_adj_ = 0.001 and 0.04 of random slope parameters were defined by means of an iterative bootstrap. The lighter lines and dots indicate mouse-specific longitudinal tumor volume measures, color-coded by specific groups. Panel **a** was created with BioRender.com. **b**, Number of metastases (*y* axis) as a function of the number of days since injection (*x* axis) and group (colors) for *n* = 10 mice (points). The colored lines show the metastatic burden fit of a Poisson generalized model with group and time as predictors. Significance levels of test of equality of metastatic burden over time between groups were obtained from Wald *t-*test *P* values for group-related GLM estimates. **c**, Kaplan–Meier plot showing the survival probability (*y* axis) as a function of time (*x* axis) per group (colored lines). Dots represent the time at which observations were right-censored. The colored triangles on the *x* axis indicate the fitted median survival times per group according to a right-censored log-normal GLM fit. Significance levels of test of equality of median survival times per group were obtained from Wald *t*-test *P* values for group-related GLM estimates. **d**, H&E images of the lung metastasis presented in histology. **e**, Halo analyses were performed to analyze % metastatic burden per lobe of the lungs *n* = 12 per cohort. Significance calculated by using a Mann–Whitney *U* test at *P* = 0.03. Metadata for the plot, upper whisker—0.055274–0.073328, 0.12079–0.14077, 0.14145–0.22992, 0.17826–0.20942; lower whisker—0.01254–0.021303, 0.0068246–0.0075507, 0.007361–0.023643, 0.007361–0.023643; upper adjacent value—0.073328, 0.14077, 0.22992, 0.20942; lower adjacent value—0.01254, 0.0068246, 0.007361, 0.026232; box bounds—0.021303–0.055274, 0.0075507–0.12079, 0.023643–0.14145, 0.053781–0.17826; median—0.026325, 0.037606, 0.037254, 0.064796; 75th percentiles—0.055274, 0.12079, 0.14145, 0.17826; 25th percentiles—0.021303, 0.0075507, 0.023643, 0.023643.

Although FOXA1 can contribute to the formation of new enhancers that drive metastasis^10^, our data suggest that it has no role in primary tumor contexts. To explore and validate the potential role of FOXA1 in PDAC, we conducted an orthotopic tumor experiment (Fig. 4a). FOXA1-OE did not change primary tumor growth in vivo compared to control mice (Fig. 4a), whereas HNF4G deletion had a substantial impact on primary tumor growth. FOXA1-OE was unable to reverse the tumor growth retardation resulting from the deletion of HNF4G, suggesting that HNF4G is the dominant transcription factor in the primary tumor context, whereas FOXA1 appears to lack any tumor-promoting activity in this context. Metastasis was not observed in control EV and FOXA1-OE mice due to the rapid primary tumor onset and the ethical requirement to kill the mice before metastases were detectable.

Next, to directly explore the role of FOXA1 and/or HNF4G in metastasis, engineered human cancer cell lines (HPAF-II) were injected into the tail vein of mice to directly assess metastatic potential. FOXA1-OE increased the number of lung metastases, regardless of whether HNF4G was present or not (Fig. 4b). This confirms that FOXA1 does indeed promote metastasis as previously suggested^10^, but it does not influence primary tumor growth (Fig. 4a). The survival data from the metastatic experiment confirmed that overexpressed FOXA1 substantially shortens overall survival, but this was substantially blunted in the absence of HNF4G (Fig. 4c). Metastatic burden to the lungs was substantially more in the FOXA1-OE and HNF4G-KO + FOXA1-OE contexts (Fig. 4d,e). Interestingly, when FOXA1 is OE in an HNF4G-KO context, these mice had extended survival, because of the HNF4G depletion, although they had widespread and substantial metastasis (Fig.4b,d,e). These data suggest that both transcription factors contribute to overall survival from PDAC, with FOXA1 mediating metastatic potential and HNF4G mediating primary growth, and possibly growth at secondary sites as well.

### HNF4G restricts the transcriptional activity of FOXA1

**Fig. 5:**
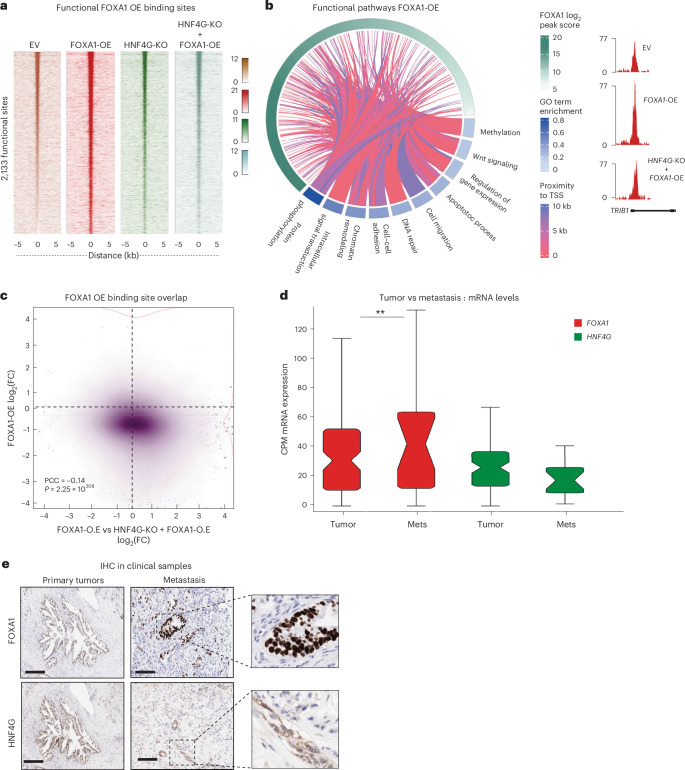
Functional FOXA1 binding in the absence of HNF4G. **a**, ChIP–seq heatmap represents coverage intensity in EV, FOXA1-OE, HNF4G-KO and HNF4G-KO + FOXA1-OE at 2,133 functional sites. **b**, Complex visualization of functional connection between FOXA1 occupancy and gene ontology groups in proximity to the functional binding sites. Color gradient reflects strength of the functional links (chr8: 125,386,596–125,482,166). **c**, Correlation of FOXA1 ChIP–seq occupancy and ATAC–seq locations in the FOXA1-OE model. The plot shows log_2_-fold changes in mapping densities between FOXA1 ChIP–seq and FOXA1-OE ATAC–seq. Correlation coefficient and *P* values have been calculated using the Pearson correlation test. **d**, PanCuRx dataset was interrogated to compare primary tumor and metastatic samples. Box plot represents the mRNA expression levels of FOXA1 and HNF4G mRNA from patient samples. The samples were ordered HNF4G primary tumor, HNF4G mets, FOXA1 primary tumor and FOXA1 mets. The samples were statistically tested using one-sided two-sample *t* test (*P* = 0.02). Samples sizes considered for these analyses include primary tumors, *n* = 105 (classical subtype); metastasis, *n* = 20. Plot metadata, upper whisker—8.2729–14.984, 6.3388–9.1325, 11.712–25.419, 14.303–29.707; lower whisker—0–2.9732, 0–2.2442, 0–2.3562, 0.37089–3.8985; upper adjacent value—14.984, 9.1325, 25.419, 29.707; lower adjacent value—0, 0, 0, 0.37089; box bounds—2.9732–8.2729, 2.2442–6.3388, 2.3562–11.712, 3.8985–14.303; median—5.7973, 4.1536, 6.8413, 10.395; 75th percentiles—8.2729, 6.3388, 11.712, 14.303; 25th percentiles—2.9732, 2.2442, 2.3562, 3.8985. ** *P* ≤ 0.03. **e**, FOXA1 and HNF4G IHC staining in primary and liver metastatic samples (*n* = 5 from cohort 1). Representative images from one patient are presented. The areas on the right show nuclear staining of FOXA1 and substantial cytosolic staining of HNF4G.

**Extended Data Fig. 5:**
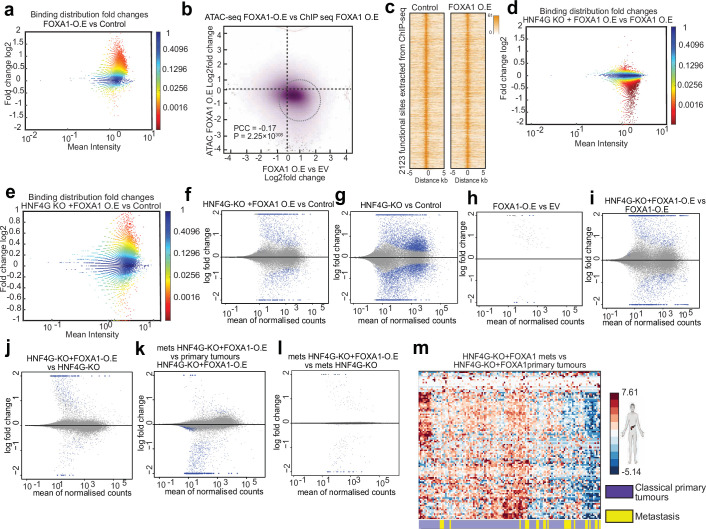
Mechanistic elucidation of HNF4G and FOXA1 context specificity. **a**. Representative MA plots with log_2_-fold change between conditions FOXA1-O.E vs control. **b**. Correlation of log_2_-fold change between FOXA1 ChIP-seq and FOXA1-OE ATAC-seq densities compared to empty vector control (EV). **c**. 2,133 sites extracted from FOXA1 ChIP-seq. Heatmap represents the open regions from ATAC-seq data between control and FOXA1-OE (n = 4). **d**. Representative MA plots with log_2_-fold change between conditions HNF4G-KO + FOXA1-OE vs FOXA1-OE. Binding distribution changes between HNF4G-KO + FOXA1-OE vs control from FOXA1 ChIP-seq data. The sites are classified as highly enriched using FDR (padj < 0.05). Color bar displays the distribution of the FDR values. **e**. Binding distribution changes between HNF4G-KO + FOXA1-OE vs control from FOXA1 ChIP-seq data. The sites are classified as highly enriched using FDR (padj < 0.05). Color bar displays the distribution of the FDR values. **f**–**l**. Normalized counts of differentially expressed genes represented as a log_2_-fold change between comparisons. Data extracted from RNA-seq analyses performed on orthotopic tumors and metastatic samples of NSG mice. Differentially regulated genes padj < 0.05, DESeq2. **m**. Differentially expressed genes from RNA-seq analysis were evaluated in samples from the PanCuRx study (130 samples). The expression pattern of the selected subset of genes derived from DeSeq2 analysis was assessed in classical subtype tumors and corresponding metastasis (mets) samples. The data were integrated with the genes from the HNF4G-KO + FOXA1-OE mets versus HNF4G-KO + FOXA1-OE primary tumors.

To date, our data suggest that HNF4G is the primary driver of tumor growth, whereas FOXA1 becomes the driver of metastasis, and together, they both contribute to tumor progression and overall survival. To understand how a functional switch occurs from HNF4G-dependent state to a FOXA1-mediated metastatic state in the transition from primary tumor growth to metastatic progression, we explored our engineered human cancer cell line xenograft models. We performed RNA-seq on the collected primary tumors from the pancreatic orthotopic experiment, as well as FOXA1 ChIP–seq and ATAC–seq to map chromatin accessibility (Extended Data Figs. 4k and 5a–c). We observed a gain of 4,940 de novo FOXA1 binding sites when FOXA1 was overexpressed compared to EV (Extended Data Fig. 5a). These sites correlated with regions that were already open and accessible, as measured by ATAC–seq (Extended Data Fig. 5b), implying that an increase in FOXA1 levels results in opportunistic binding to regions that are already euchromatic and FOXA1 cannot create new enhancers, as would be expected from a genuine PF. Unexpectedly, no differential gene expression changes were observed after FOXA1-OE (Extended Data Fig. 5f–h), despite the gain in FOXA1 binding sites. When HNF4G was silenced, however, 2,133 of these gained FOXA1 binding sites (following FOXA1-OE) became transcriptionally active (Fig. 5a,b and Extended Data Fig. 5c–e), suggesting that the presence or absence of HNF4G dictates whether FOXA1 is active at these regions or not (Extended Data Fig. 5i,j).

**Extended Data Fig. 6:**
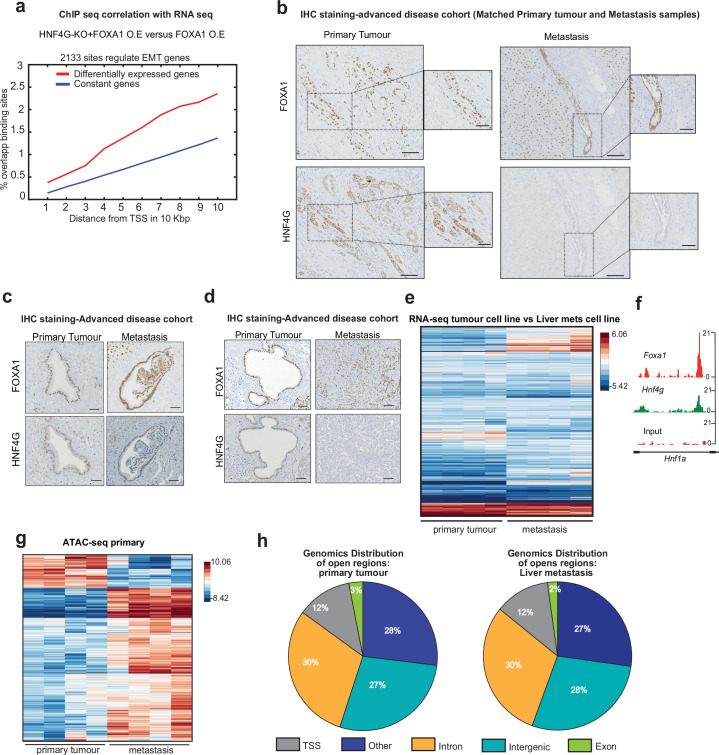
IHC validation of FOXA1 and HNF4G in two independent patient cohorts. **a**. Functional relation of FOXA1-dependent sites to gene expression. Curves correspond to the cumulative percentage of FOXA1 binding sites within a distance from the transcription start site (TSS) of either significantly up-regulated or constantly expressed genes in HNF4G-KO + FOXA1-OE. l. Model of FOXA1 and HNF4G binding between primary tumor and metastasis contexts. **b**&**c** IHC images from advanced PDAC patients (independent cohort). IHC staining of FOXA1 and HNF4G in primary tumor and liver metastases samples. Images are representative of a cohort of 28 independent patient samples. **d**. Heatmap representing the open, accessible regions extracted from ATAC-seq data comparing KPC-derived primary tumor cell lines and matched liver metastasis cell lines (n = 4). Red represents open regions; blue represents closed regions. **e**. Genomic feature distribution from ATAC-seq profiles between primary tumor cell lines and liver metastatic cell lines across four biological replicates. **f**. RNA-seq analysis was performed in KPC-derived matched primary tumor and liver metastasis cell lines (n = 4). Heatmaps highlight differentially regulated genes, padj < 0.01, using DESeq2 across four independent biological replicates. **g**. IGV (genome browser) screenshots representative of Foxa1 (red) and Hnf4g (green) binding sites in the KPC-derived isogenic cell lines.

These new FOXA1 binding sites (that are normally restricted by HNF4G) result in induced gene expression profiles associated with metastasis (Fig. 5b,c). We term these ‘HNF4G-restricted FOXA1 metastasis genes’. These HNF4G-restricted FOXA1 metastasis genes were correlated well with the gene expression profiles of metastatic patient samples (mets) when compared to a cohort of classical subtype primary tumor samples (from the PanCuRx dataset; Extended Data Figs. 5m and 6a), validating the conclusion from the preclinical models.

### HNF4G dependency to FOXA1-driven metastatic transition

Up to this point, our data suggest that HNF4G is the driver in primary contexts, and it also restricts FOXA1’s metastatic activity. We speculated that HNF4G’s ability to restrict FOXA1 could be compromised in advanced disease, where metastatic target genes become induced by FOXA1. Analysis of the human PanCuRx dataset showed a global decrease in HNF4G mRNA expression in metastatic samples (mets) relative to primary tumors, with a coincident increase in FOXA1 expression (Fig. 5d). We also conducted IHC of HNF4G and FOXA1 from primary tumors and relatively rare metastatic human tissue (two independent cohorts). In primary tumors, we observed clear expression of HNF4G and FOXA1, which was restricted to the epithelial compartment and predominantly exhibited nuclear expression (Fig. 5e and Extended Data Fig. 6b–d). When we assessed the metastatic patient samples, we observed a consistent expression of FOXA1. However, in two independent cohorts, we observed either decreased HNF4G protein expression or cytoplasmic HNF4G staining in metastases, which was not observed in primary tumor samples (Fig. 5e and Extended Data Fig. 6b–d). Our data suggest that multiple mechanisms exist for a tumor to inhibit HNF4G activity, allowing FOXA1 to become active and drive metastatic spread.

### Context-specific genomic spatial re-organization

**Fig. 6:**
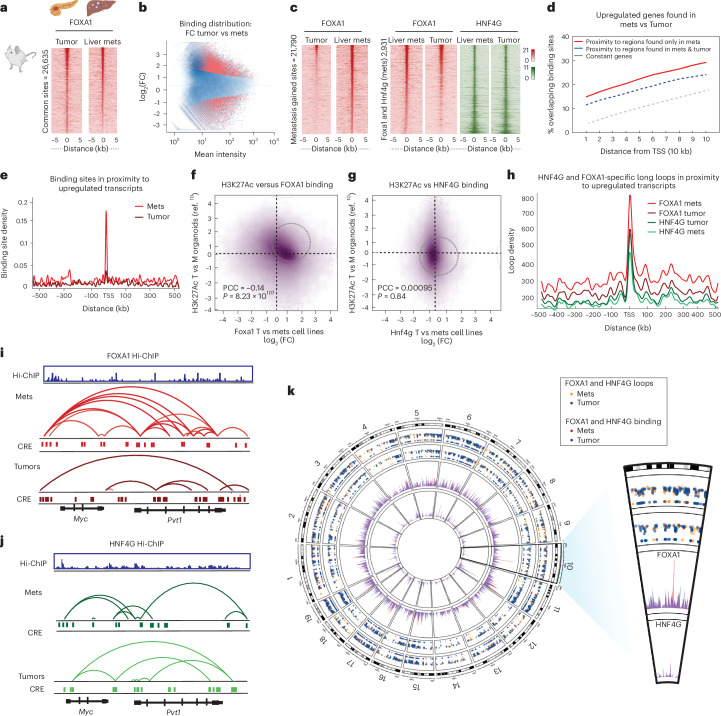
Context-specific genomic spatial re-organization mediates FOXA1-driven metastasis. **a**, FOXA1 ChIP–seq performed in KPC mouse-derived primary tumor cell line and matched liver metastasis (mets) cell line. Heatmaps illustrate normalized coverage density at common ChIP–seq sites. Panel **a** was created with BioRender.com. **b**, Foxa1 binding sites that were either common to both tumor and liver mets, tumor-unique or liver metastasis-unique (gained). The data have been analyzed using negative binomial GLM as a part of the DiffBind package at *P*_adj_ < 0.05 threshold. **c**, Heatmaps represent occupancy of Foxa1 and Hnf4g at common sites in the metastatic cell line model, ordered by Foxa1 intensity. **d**, The plot illustrates the relationship in cumulative numbers of Foxa1 binding sites located within the 10-kb linear scale from TSS of differentially upregulated and constantly expressed genes from the RNA-seq data. **e**, Difference in landscape of Foxa1 binding density across genomic ranges relative to TSS of induced genes, between mets and primary cell lines. **f**,**g**, Correlation of log_2_-fold changes between H3K27Ac (T versus M organoid models extracted from ref. ^10^) with ChIP–seq of Foxa1 (**f**) and Hnf4g (**g**) (mets versus primary tumor cell lines). **h**, Difference in Foxa1-induced chromatin loops in proximity to upregulated transcripts across genomic ranges relative to TSS, comparing mets and primary cell lines. **i**,**j**, An example of Foxa1 (**i**) and Hnf4g (**j**) associated Hi-ChIP loops in mets and tumors. Foxa1 and Hnf4g binding at CREs in mets and tumor (red and green). The example shows looping between the *c-Myc* enhancers in mets and Foxa1 and Hnf4g binding is shown in blue. **k**, Combined genome-wide view of functional relationships between ChIP and Hi-ChIP data in the context of mets and tumor. Both FOXA1 and HNF4G ChIP were mapped to the mouse GRC mm10 genome and represented as tracks. Corresponding Hi-ChIP loops are shown as scatters in sectors mapped to chromosomes in the ideogram. TSS, transcription start sites.

To support our human clinical and preclinical data, we took an orthogonal approach and exploited the autochthonous KPC mouse model^28^. We derived and validated cell line models from KPC primary tumors or from matched liver metastases, permitting a comparison of isogenic matched cell line models (Extended Data Fig. 6e–h). There was a substantial gain of global Foxa1 binding in the liver metastasis (mets) cell line compared to the primary tumor cell line (Fig. 6a), with a total of 21,790 metastasis-specific gained Foxa1 binding sites (Fig. 6b,c). The ability of elevated Foxa1 to associate with these enhancers resulted in the induction of adjacent genes (Fig. 6d and Extended Data Fig. 6e,f), which were enriched in pathways associated with metastasis. We exploited the published H3K27Ac ChIP–seq data derived from mouse organoid models^10^ and demonstrated that the Foxa1 binding observed specifically in our metastasis-derived KPC mouse cell line model was located at the same genomic regions where H3K27Ac was mapped in the published mouse metastatic organoids (Fig. 6f,g).

**Fig. 7:**
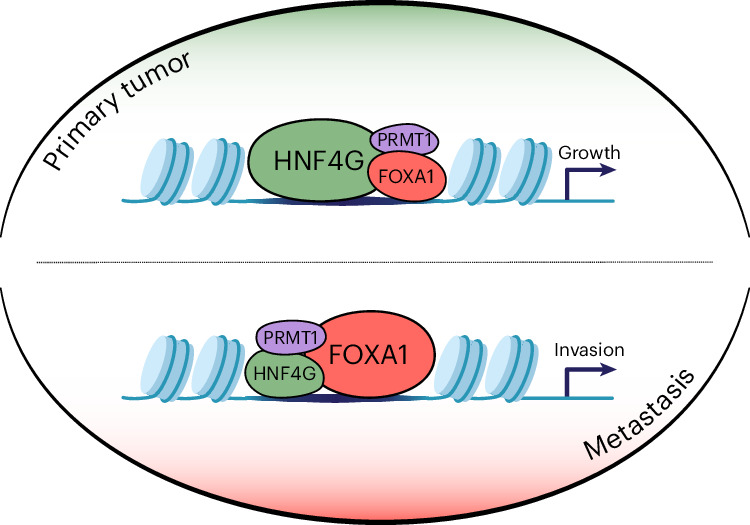
Model of HNF4G and FOXA1 in primary tumor and metastatic context. The top scenario represents primary tumor contexts, where HNF4G is the dominant transcription factor, required for chromatin accessibility, gene expression and cell growth. The bottom scenario represents metastatic contexts, where FOXA1 becomes functional and the role of HNF4G is diminished. The switch from HNF4G dependency to FOXA1 dependency mediates the molecular transition from primary tumor growth to metastasis.

**Extended Data Fig. 7:**
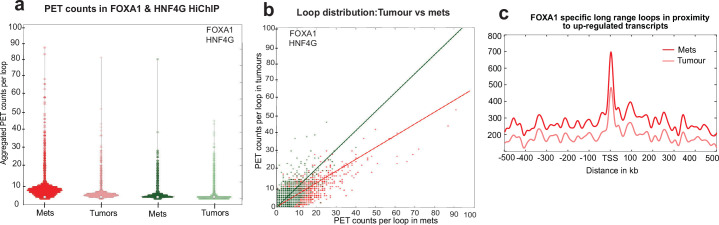
FOXA1-induced context-specific chromatin looping. **a**. Distribution of loop-supporting normalized PETs across samples represented as violin plots. **b**. Scatter plot shows correlation in looping in tumor versus mets in the Foxa1 and Hnf4g Hi-ChIP samples. **c**, Density landscape of Foxa1-associated chromatin loops in proximity to up-regulated transcripts in mets versus tumor cell lines across genomic ranges relative to TSS.

We hypothesized that Foxa1 could promote preferential enhancer–promoter interactions in the metastatic context. To this end, we performed Hi-ChIP in the two isogenic mouse cell lines derived from the KPC mice and found that Hnf4g and Foxa1 were associated with chromatin looping (Fig. 6h,i,j). Hnf4g Hi-ChIP revealed elevated and preferential long-range looping in the primary tumor context compared to metastasis (Fig. 6h,j and Extended Data Fig. 7a,b). In contrast, Foxa1 Hi-ChIP revealed a global gain of enhancer–promoter looping events specifically in the metastatic mouse cell line (Fig. 6h,i and Extended Data Fig. 7a,b). These data implicate FOXA1 (Foxa1) in metastasis-specific chromatin looping interactions that culminate in the metastasis-specific gene expression program (Fig. 6i,k and Extended Data Fig. 7c). Therefore, while HNF4G is the dominant transcription factor in primary tumor contexts, a switch occurs in advanced disease, resulting in engagement of FOXA1 to enhancers that create spatial genomic re-organization and ultimately in induction of metastasis-specific gene targets.

## Discussion

Recent studies have implicated certain transcription factors as signature genes of specific PDAC subtypes and have linked several of these to disease progression^2,5,22^. Although transcription factors in the past have been considered ‘undruggable’, NR transcription factors constitute realistic drug targets due to the presence of a ligand-binding pocket in the protein. To this end, our new insight reveals the NR HNF4G as a key driver in the primary tumor growth. Currently, HNF4G-targeted drugs do not exist and HNF4G is an orphan NR with no known endogenous ligand. One possibility is that HNF4G might lack an endogenous ligand and could have evolved as an NR-related transcription factor that does not functionally require the conserved ligand-binding domain. Alternatively, there could be an endogenous ligand for HNF4G that is a noncanonical compound. In support of this, X-ray crystallographic structures of HNF4G have revealed a common long-chain fatty acid, palmitic acid, occupying the ligand-binding domain pocket of HNF4G in these structures and palmitic acid has previously been shown to influence PDAC tumor growth and metastatic potential^29,30^.

The family members of HNF transcription factors have been previously implicated as fundamental regulators of gene expression programs in bowel disease, colorectal cancer and PDAC^2,11,14^. HNF1A deficiency has been shown to promote PDAC and is also associated with the squamous subtype, through the recruitment of the demethylase enzyme KDM6A^31^. A study in ref. ^11^ reported BMP/SMAD signaling as a mechanism to stabilize enterocyte identity via HNF4 transcription factors. Specifically, for PDAC, a study in ref. ^12^ observed that the loss of these endoderm-associated transcription factors (that is, HNF4A and GATA6) could result in altered metabolic profiles of cells associated with the squamous lineage. We did not find a substantial role for HNF4A in the classical subtype; however, a role for HNF4A may exist in different stages of the disease, particularly in late-stage disease.

Surprisingly, little is known about the normal physiological role of HNF4G, very little information exists linking it to disease, and gene KO data suggest that it is not an essential gene for developmental processes and mouse viability. In contrast to HNF4G, FOXA1 has been previously implicated as a driver of PDAC metastasis, with elevated FOXA1 creating de novo enhancers associated with metastasis^10^. FOXA1 is the archetypal PF^6,32^, and its functional role as the adaptor between chromatin and NR transcription factors in breast and prostate cancer is well-established^33,34^. In both hormone-dependent breast and prostate cancers, FOXA1 forms a triumvirate with a GATA protein and an NR (GATA3 and ER in breast cancer; GATA2 and AR in prostate cancer) and is the foundational protein in these cancers^35,36^ due to its ability to create new enhancer elements^37^. However, the role of FOXA1 in primary PDAC disease was in stark contrast to what is observed in breast and prostate cancer^35,38^. FOXA1 could bind to active enhancers in PDAC (in primary tumor tissue and the various models) at the same places as HNF4G; we could not observe a role for FOXA1 in mediating chromatin accessibility, gene expression or cell viability in primary tumor contexts. This suggests a nonpioneering role for FOXA1 in PDAC and a dominant role for HNF4G in this subtype of the disease.

We identified the core transcription factors that make up the lineage-defining factors in the classical subtype of PDAC. In the primary tumor context, HNF4G was the dominant transcription factor that drives tumor growth, while also negatively regulating FOXA1 transcriptional activity. During the transition to metastasis, however, we observed a substantial decrease in HNF4G:FOXA1 ratios and, mechanistically, we could show that when HNF4G is depleted, FOXA1 becomes active and is associated with chromatin loops that drive expression of a distinct gene expression program associated with metastatic potential^10^. Overall survival in the classical subtype is dictated by the proliferative activity of HNF4G and the metastatic activity of FOXA1.

## Methods

### Clinical material

Matched flash frozen and formalin-fixed paraffin-embedded tissue (FFPE) samples from Whipple surgical samples undergoing surgical resection at the Cambridge University Hospitals were sectioned according to institutional protocols.

This study was approved by the East of England—Cambridgeshire and Hertfordshire Research Ethics Committee and was conducted in compliance with Good Clinical Practice, local regulatory requirements and the legal requirements for CAMPAN (08/H0306/32). All patients provided written informed consent. All procedures performed in studies involving human participants were in accordance with the ethical standards of the institutional and/or national research committee and with the 1964 Helsinki Declaration and its subsequent amendments or comparable ethical standards. Release of data was also pseudo-anonymized as per the UK Human Tissue Act regulations.

Histological staining of FOXA1 and HNF4G in stage IV primary tumors and liver metastases was performed according to the recommendations of the local ethics committee of the Medical Faculty of the University of Duisburg—Essen (approval 23-11451-B0). Clinical data were obtained from archives and electronic health records. All patients provided written informed consent.

### Cell lines

AsPC-1, HPAF-II, BXPC-3, Panc-1, MiaPaca (ATCC), KPC-derived cell lines and CRISPR-modified cell lines were grown in DMEM (Gibco, 41966-029), supplemented with 10% FBS (Gibco, 10500-064). Cells were routinely genotyped by short-tandem repeat genetic profiling using the PowerPlex 16HS cell line panel and analyzed using the Applied Biosystems GeneMapper ID (v3.2.1) software by the external provider Genetica DNA Laboratories (LabCorp Specialty Testing Group). Cells were tested using MycoProbe Mycoplasma detection kit (R&D) before experiments every 6 months. All cell lines were grown at 37 °C.

### Generation of HNF4G-KO by CRISPR

Three CRISPR guides were designed against exons 3 and 4 of HNF4G (ENSG00000164749). The three sequences are as follows: (1) 196 (exon 4)—CATCCCCTCCATTAACACAC, (2) 197 (exon 3)—GTGTTGTTGACAAGGACAAA and (3) 198 (exon 3)—TTAAGAAAGTGTTTTAGAGC.

HPAF-II cells were electroporated (Amaxa 4D nucleofector, Lonza) with 5-μg TrueCut spCas9 protein V2 (Invitrogen) and 100 pmol of single guide RNA (Synthego), and used program DS137 and P3 nucleofector solution (Lonza). A cell pellet was taken 3 days after electroporation, and genomic DNA (gDNA) was extracted from each pool. Exon 3 or 4 of HNF4G was amplified by PCR (Q5 polymerase, NEB). Amplicons were subjected to Sanger sequencing and analyzed using the Synthego ICE web tool to calculate the percent editing. To generate clonal cells, HNF4G edited pools were single cell cloned into 96-well plates. gDNA was extracted from each clone and the editing analysis was performed similarly to the edited pool. Three individual clones were confirmed to be KOs and pooled together to make an HNF4G clonal pool. Three clones with heterozygotic frameshift mutations were used for subsequent experiments. The target frameshift mutations in these three clones were validated by Sanger sequencing. The HNF4G^−/−^ pool of clones were short-tandem repeat genotyped and mycoplasma tested as described under ‘Cell lines’.

### siRNA-mediated knockdown

HPAF-II cells were transfected with ON-TARGET plus SMARTPools (Dharmacon) FOXA1 (L-010319-00-0020), ON-TARGET plus SMARTPools (Dharmacon) HNF4G (set 1 L-003407-00-0020 and set 2 stealth sirna-HSS104886, HSS179284 and HSS104885), HNF4A (stealth sirna-HSS140900 and HSS140901) and RNAiMAX (Invitrogen, 13778-150). A nontargeting pool (D-001810-10-20) was used as a control.

### RIME processing

RIME on cell lines was performed as described previously^39^. Briefly, cells were cross-linked at room temperature by incubating with 2 mM di(N-succinimidyl) glutarate (DSG) for 20 min followed by 1% formaldehyde for 10 min before quenching in 0.1-M glycine for 10 min. Cells were washed twice in cold PBS and collected in cold PBS containing protease and phosphatase inhibitors(Roche, Complete, 05056489001) and Halt inhibitors (Thermo Fisher Scientific, 78427). Cross-linked cells were incubated at 4 °C with lysis buffer (LB1; 50 mM Hepes–KOH, pH 7.5, 140 mM NaCl, 1 mM EDTA, 10% glycerol, 0.5% NP-40/Igepal CA-630, 0.25% Triton X-100) for 10 min followed by 5 min in LB2 (10-mM Tris–HCl, pH 8.0, 200 mM NaCl, 1 mM EDTA, 0.5 mM EGTA) before resuspending in LB3 (10 mM Tris–HCl, pH 8, 100 mM NaCl, 1 mM EDTA, 0.5 mM EGTA, 0.1% Na-deoxycholate, 0.5% N-lauroylsarcosine). Chromatin was sonicated using the Bioruptor Plus (Diagenode) for 15 min (30-s on/30-s off) to generate fragments of around 100–800 bp. Chromatin was immunoprecipitated overnight using protein A Dynabeads (Invitrogen; used for rabbit antibodies) or protein G Dynabeads (Invitrogen; used for goat antibodies) conjugated to specific antibodies against HNF4G (Atlas, HPA005438), HNF4A (Cell Signaling Technology, 3113S), PRMT1 (Atlas, HPA072136), GATA6 (Cell Signaling Technology, 5851; R&D Systems, AF1700) and FOXA1 (Abcam, ab5089). Beads were washed ten times in RIPA buffer (50 mM, pH 7.6, 1 mM EDTA, 0.7% Na-deoxycholate, 1% NP-40, 0.5 M LiCl) followed by two washes in AMBIC (100 mM ammonium hydrogen carbonate). Washed beads were frozen at −20 °C.

### Sample preparation liquid chromatography–mass spectrometry analysis and data processing

Pull-down samples were digested, and peptides were cleaned with C18 spin columns as described previously^39^. Peptides were analyzed in the Dionex UltiMate 3000 UHPLC system coupled with the Q-Exactive HF (QE-HF) or QE (Thermo Fisher Scientific) mass spectrometers. For peptide separation, the EASY-Spray analytical column (75 μm × 25 cm, C18, 2 μm, 100 Å) was used for multistep gradient elution. The full scans were performed in the Orbitrap over the range of 400–1,600 *m*/*z* at 60k (QE-HF) or 70k (QE) resolution. For MS2, the ten most intense precursors were selected with resolution of 30k (QE-HF) or 17.5k (QE). The HCD tandem mass spectra were processed with the Sequest HT search engine in Proteome Discoverer 1.4 or 2.2 software. For downstream statistical analysis, the Perseus software was used^40^.

### ChIP–seq

Clinical samples and cell lines ChIP–seq on tumor material were performed essentially as described previously^41^. Briefly, flash-frozen pancreatic cancer or normal adjacent tissue from patients was cryosectioned into 30-μm sections, cross-linked by 2 mM DSG for 45 min, total together with 1% formaldehyde for the last 20 min and quenched by 0.1 M glycine for 10 min at room temperature. Chromatin was immunoprecipitated, washed and processed for sequencing. ChIP–seq was performed on three or four independent biological replicates. Chromatin was prepared from both cell lines/tumors and immunoprecipitated as described for RIME above. Chromatin was eluted and decrosslinked by incubating overnight at 65 °C in elution buffer (50 mM Tris–HCl, pH 8.0, 10 mM EDTA, 1% SDS). Samples were treated with (20 ng ml^−1^) RNase A for 30 min–1 h followed by proteinase K (200 ng ml^−1^) for 1–2 h before DNA was purified by phenol-chloroform extraction. Purified DNA was subjected to library preparation using the SMARTer ThruPLEX DNA-seq Kit (Takara Bio, R400676) and DNA HT Dual Index Kit—96N Set A (Takara Bio, R400660) followed by next-generation sequencing to reach approximately 30 million reads per sample.

### ChIP–seq analyses

A total of 50-bp single-end reads were mapped to the hg38 genome using bowtie2 (v2.2.6). Aligned reads with mapping quality <5 were filtered out. The read alignments from all replicates were combined into single library and peaks were called with MACS2 (version 2.1.1.2016) using input sequences as a background control. The peaks that absorbed statistically significant tag density from all replicates were selected for downstream analysis. MEME (version 4.9.1) was used to detect known and discovered new binding motifs among tag-enriched sequences. For visualizing tag density and signal distribution heatmap, the read coverage in a window of +/−2.5 or 5 kb of the peak midpoint was generated using a bin size of 1 of 100 of the window length. Differential binding analysis (DiffBind) was performed as described previously^42^.

### ChIP–seq enrichment near regulated genes

The cumulative numbers of ChIP–seq binding sites located within the 10-kb linear scale from the transcription start sites of substantially upregulated genes were determined. As a background control, binding sites near an equal number of randomly expressed genes revealed by the RNA-seq analyses were tested in 100 permutations. The fraction of binding sites near these groups of genes was then visualized as curves, including the s.d. for the random constitutively expressed control genes as error bars. For the circular visualization of functional relations between the ChIP–seq and corresponding gene network, we used the circlize package in R (version 4.4.36). Networking analysis of predicted gene associations was performed using the STRING application plugin with Cytoscape (version 3.10.3).

### RNA-seq

Total RNA was purified using RNeasy kit (Qiagen, 74106) and mRNA libraries were prepared using the stranded mRNA library preparation kit (Illumina, 20020595) and IDT for Illumina—TruSeq RNA UD Indexes (20022371). Samples were subjected to Illumina next-generation sequencing to reach around 30 million reads per sample. Four biological replicates were conducted for all RNA-seq experiments.

### RNA-seq analyses

A total of 50 bp single-end reads were aligned to the human genome (GRCh37) or mouse genome (GRCm38) using STAR (version 2.6.1a). Differentially expressed genes were identified based on the negative binomial distribution using the DESeq2 package in R (version 1.14.1). Heatmap visualization, clustering and various plotting functions were used from statistical and bioinformatic modules in framework.

### ATAC–seq

HPAF-II cells with HNF4G-KO and control, alongside FOXA1-OE with empty vectors (EVs), were used for ATAC–seq protocol. Omni-ATAC–seq was performed as described previously^43^. ATAC–seq resuspension buffer (RSB) was prepared as follows: for 50 ml of the buffer, 500 μl of 1 M Tris–HCl, pH 7.4, 100 μl of 5 M NaCl, 150 μl of 1 M MgCl_2_ and 49.25 ml sterile water were added. A total of 50 μl cold ATAC–RSB containing 0.1% NP-40, 0.1% Tween-20 and digitonin 0.01% (Promega, G9441) was added to 50,000 viable cells and pipetted up and down thrice. The cells were incubated on ice for 3 min to lyse the cells and washed with 1 ml of cold ATAC–RSB containing 0.1% Tween-20, but without NP-40 and digitonin. The pellet was resuspended in 50 µl transposition mix (25 µl 2× Tagment buffer, 2.5 µl transposase—100 nM final; Illumina Tagment DNA Enzyme and Buffer Small Kit, 20034197—16.5 µl PBS, 0.5 µl 1% digitonin, 0.5 µl 10% Tween-20, 5 µl H_2_O) and the reaction was incubated at 37 °C for 30 min in a thermomixer (1,000 rpm mixing). Zymo DNA Clean and Concentrator-5 kit (D4014) was used to clean up the reaction and DNA was eluted in 21 μl elution buffer and stored at −20 °C until amplification. A total of 20 μl of the product was used for the following PCR. Pre-amplification was performed for five cycles using 1 μl of i5 primer, 1 μl of i7 primer, 25 μl 2× Q5 HotStart NEBNext master mix (M0494L) and 20 μl transposed/cleaned-up sample. Thermocycler conditions were as follows: 72 °C for 5 min and 98 °C for 30 s, followed by five cycles of 98 °C for 10 s, 63 °C for 30 s and 72 °C for 1 min, then held at 4 °C. Using 5 μl (10%) of the pre-amplified mixture, 15 μl qPCR was run to determine the number of additional cycles needed—3.85 μl sterile water, 0.2 μl Nextera XT Index kit (24 indexes, FC-131-1001) i5 primer, 0.2 μl i7 primer, 0.75 μl 20× EvaGreen (in DMSO), 5 μl 2× NEBNext master mix and 5 μl pre-amplified sample. Cycling conditions wer as follows: 98 °C for 30 s, followed by 20 cycles of 98 °C for 10 s, 63 °C for 30 s and 72 °C for 1 min. After qPCR amplification, the amplification profiles were analyzed to determine the required number of additional cycles for amplification. Samples were size selected and purified using Ampure XP beads. Furthermore, NGS was performed using Illumina NovaSeq 6000 with SP flow cell system with 50-bp paired end.

### Western blot

Cells were washed twice in cold PBS and collected in RIPA buffer (Pierce, 89901). Whole-cell extract was sonicated using the Bioruptor Plus (Diagenode) for 2 min (30-s on/30-s off) to degrade the DNA, quantified by Direct Detect spectrometer Millipore) and run on NuPAGE 4–12% Bis–Tris gels (Invitrogen). Proteins were transferred to a membrane by iBlot2 (Invitrogen), and the membrane was then blocked with Odyssey TBS blocking solution (Li-Cor, 927-50000) and incubated with antibody overnight at 4 °C. After washing, the membrane was then incubated with appropriate secondary antibodies (Li-Cor) and developed using the Odyssey CLx Imaging System (Li-Cor). Primary antibodies were as follows: FOXA1 (Abcam, ab23738), HNF4G, HNF4A, GATA6 (Cell Signaling Technology, 5851; monomethyl arginine, 8015; Vinculin, 13901; β-actin, 4970); asymmetric dimethyl (arginine, 13522; Sigma-Aldrich, A5441). Secondary antibodies (Invitrogen) were as follows: goat anti-rabbit (926-32211, 926-68071), goat anti-mouse (926-32210, 926-68070) and donkey anti-goat (926-32214).

### IHC staining

Five tumors per treatment arm were fixed in formalin for 24 h and subsequently transferred to 70% ethanol and paraffin embedded. Sections were cut, dewaxed, rehydrated and subsequently stained with antibodies on Ventana (Roche) using the fully automated Ventana Discovery ULTRA (Roche) with Ventana solutions. Paraffin sections were pretreated with heat using standard conditions (40 min) in CC1 solution. The primary antibody was applied and incubated for specific times (listed below). After incubation with a rabbit Impress HRP antibody (Vector Laboratories), chromogenic revelation was performed with ChromoMap DAB kit (Roche). Thus, IHC staining for FOXA1, HNF4G and PRMT1 was performed with antibodies described above. Slides were scanned using an Aperio AT2 (Leica) at ×20 magnification (resolution = 0.5 μm per pixel).

### Hi-ChIP

Hi-ChIP was performed using a previously published protocol. Once the ligation step was completed by adding 50 µl of 1 U µl^−1^ T4 DNA ligase (Invitrogen, 15224-025) and incubating for 4 h at room temperature with gentle mixing on shaker (lowest speed), the chromatin was spun down, and the ChIP–seq protocol as described above was followed.

### Hi-ChIP data processing

Hi-ChIP paired-end data processed using the HiC-Pro pipeline, followed by the Hichipper algorithm, reads were aligned to mm10 genomes using the HiC-Pro pipeline. Default settings were used to remove duplicate reads, assign reads to Dpn2 restriction fragments, filter for valid interactions and generate binned interaction matrices^44,45^.

### Animals

All experiments were performed in accordance with the animal project licenses P40BD8F30, P1919999D and PP79993249 in accordance with Home Office guidelines.

NSG mice (~8 weeks old) were obtained from Charles River Laboratories, kept in pathogen-free conditions on a 12-h light/12-h dark cycle and allowed to acclimatize for a period of 7 days before any surgery. Mice were grouped, housed with environmental enrichment, fed on a maintenance diet (PicoLab) and housed with a room temperature of +22 °C/−2 °C and humidity of +55%/−10%. For survival studies, humane endpoints were used with the loss of 15% of pre-implantation weight, body condition score <2.0. Grimace scale (MSG > 1.2/2) scoring was adhered to alongside other clinical signs. All mice were randomly assigned to cohorts as necessary, and analyses were performed in a blinded manner.

### In vivo orthotopic transplantations

Pancreatic orthotopic injections were performed as described previously^46^. HPAF-II cells were suspended in 50% Matrigel/PBS and injected into the tail of the pancreas (2.5 × 10^5^ cells in 50 µl), with the exception of the FOXA1-OE study, which used 1 × 10⁴ cells per cohort. (Fig. 3g). Mice were allowed to recover from surgery before the onset of routine palpations (weekly from 2 weeks postsurgery) to determine the presence of any pancreatic masses. Once confirmed, mice were serially imaged (weekly) using the Vevo 2100 ultrasound imaging platform (Visual Sonics; 13–24-MHz transducer) to monitor pancreatic tumor progression until clinical endpoint. Images of all tumors were acquired at a minimum of two different orientations. All study images were randomized and blinded before calculating tumor volumes using 3D volume analysis software (Vevo LAB, version 3.1.1) on images taken at the same orientation for each mouse throughout the study, whenever possible. Mice were randomized into four groups (*n* = 9 mice for control vehicle and HNF4G-KO vehicle arms; *n* = 14 for control + GSK3368715 arm and *n* = 15 for HNF4G-KO + GSK3368715 arm). At 2 weeks postsurgery, mice were treated with either vehicle (control, Vetivex saline) or GSK3368715 (75 mg kg^−1^) by oral gavage (5 ml kg^−1^), using a dosing regimen of 5 days on/2 days off until clinical endpoint was reached.

### Lung metastasis assay

Pancreatic cancer cells were injected into the tail vein of NSG mice (*n* = 12 per group). HPAF-II cells were prepared as a suspension (in PBS) and injected (0.5 × 10^6^ cells in 100 µl) into the tail vein of NSG mice. Mice were monitored daily for signs of ill health or body weight loss for the first 7 days postinjection and then, at routine, periodic intervals until clinical endpoints were reached, whereby necropsies were performed and metastatic burden assessed, complemented by histological analyses. Mice were allowed to recover from surgery for 2 weeks before the onset of routine health checks. Mice were monitored for clinical endpoints, and necropsies were performed to assess metastatic burden by histology.

### Statistical analysis

#### Survival analyses

In Figs. 1c, 2b and 4a, we displayed the survival probability of each group as a function of time by means of Kaplan–Meier plots. Due to the absence of right censoring in the data considered in Fig. 2b, we used a two-sided two-sample Mann–Whitney–Wilcoxon test (function Wilcox test of R stats package) to test if the median of the difference between a sample of each group is different from 0. In Fig. 1c, due to the presence of right censoring in the TCGA group, we used a log-rank test (function survdiff of the R survival package) to assess whether there is a difference between the two survival curves. In Fig. 4a, due to the absence of right censoring, we considered one-sided Welch tests (function *t* test of the R stats package) to assess whether the average log survival times of each group compared to the reference (control–vehicle) were significant, and the *P* values by means of a Bonferroni multiplicity correction. Sensitivity analyses, considering Wald *t-*tests based on a log-normal model and Mann–Whitney–Wilcoxon tests, led to the same conclusions.

#### Growth curves

In Figs. 2c and 4b,e, we used parametric linear mixed models to fit tumor sizes of mice on the cube root (Figs. 2c and 4b) and log plus 1 (F3k) scale as a function of groups and time. These transformations were selected to obtain a linear relationship between tumor size and time on the transformed scales and to tame heteroscedasticity. In each analysis, groups (considered as factor with a reference group), time (in days) and groups interacting with time were used as fixed effects. Random intercepts (Figs. 2c and 4b) and slopes (Figs. 2c and 4e) were used to take the longitudinal within-mouse tumor size dependence into account.

In Fig. 2c, the difference in growth between the control and HNF4G-KO groups was assessed by means of a likelihood ratio test comparing the likelihood (of the maximum likelihood estimator) of a restricted model assuming the same average growth for both groups and a full model that allows both groups to have a different average growth.

In Fig. 4e, given the delayed growth of the HNF4G groups compared to the control ones, we opted for a piecewise mixed model that allows for the modeling of this time lag, whose value was obtained by minimizing the Akaike criterion. Inference for the parameters related to the growth time lag between the HNF4G and control groups, as well as the difference in growth between each group and HNF4G-KO-GSK, was based on Wald *z* tests, considering a multiplicity correction for parametric models (function glht of the R multcomp package).

In Figs. 2c and 4e, models were fitted using standard R routines (function lme of the nlme R package). In Fig. 4e, due to the combination of small sample size per group and the presence of left truncation, we used an estimator based on the iterative bootstrap. This allows us to correct for a potential left-truncation bias and to perform a finite sample correction for the parameters. Its initial estimator was a random slope model with the fixed effects described above and a linear interpolation of left-truncated observations. Two-sided inference for the target parameters considered a Bonferroni correction for multiple testing.

### Reporting summary

Further information on research design is available in the Nature Portfolio Reporting Summary linked to this article.

## Online content

Any methods, additional references, Nature Portfolio reporting summaries, source data, extended data, supplementary information, acknowledgements, peer review information; details of author contributions and competing interests; and statements of data and code availability are available at 10.1038/s41588-025-02389-7.

## Supplementary information

Reporting Summary

Supplementary Table 1 Supplementary Table 1.

## Source data

Source Data Extended Data Figs. 2–4 Source data for Extended Data Figs. 2d,i, 3e and 4b,f.

## Data Availability

All ChIP–seq, Hi-ChIP, ATAC–seq and RNA-seq data have been deposited at Gene Expression Omnibus and can be accessed at GEO submission (GSE245734). Please access data using the link https://www.ncbi.nlm.nih.gov/geo/query/acc.cgi?acc=GSE245734. All proteomic data have been deposited at PRIDE and can be accessed at PXD045980. Source data are provided with this paper.

## Code availability

Refer to https://github.com/igorchern/jclabcode (10.5281/zenodo.16925967)^47^ and reporting summary for code information.

## References

[1] Biankin, A. V. et al. Pancreatic cancer genomes reveal aberrations in axon guidance pathway genes. *Nature* **491**, 399–405 (2012). 23103869 10.1038/nature11547 PMC3530898

[2] Bailey, P. et al. Genomic analyses identify molecular subtypes of pancreatic cancer. *Nature* **531**, 47–52 (2016). 26909576 10.1038/nature16965

[3] Humphris, J. L. et al. Hypermutation in pancreatic cancer. *Gastroenterology* **152**, 68–74 (2017). 27856273 10.1053/j.gastro.2016.09.060

[4] Waddell, N. et al. Whole genomes redefine the mutational landscape of pancreatic cancer. *Nature* **518**, 495–501 (2015). 25719666 10.1038/nature14169 PMC4523082

[5] Moffitt, R. A. et al. Virtual microdissection identifies distinct tumor- and stroma-specific subtypes of pancreatic ductal adenocarcinoma. *Nat. Genet.* **47**, 1168–1178 (2015). 26343385 10.1038/ng.3398 PMC4912058

[6] Cirillo, L. A. et al. Binding of the winged-helix transcription factor HNF3 to a linker histone site on the nucleosome. *EMBO J.* **17**, 244–254 (1998). 9427758 10.1093/emboj/17.1.244 PMC1170375

[7] Cirillo, L. A. et al. Opening of compacted chromatin by early developmental transcription factors HNF3 (FoxA) and GATA-4. *Mol. Cell* **9**, 279–289 (2002). 11864602 10.1016/s1097-2765(02)00459-8

[8] Pomerantz, M. M. et al. The androgen receptor cistrome is extensively reprogrammed in human prostate tumorigenesis. *Nat. Genet.* **47**, 1346–1351 (2015). 26457646 10.1038/ng.3419 PMC4707683

[9] Hurtado, A., Holmes, K. A., Ross-Innes, C. S., Schmidt, D. & Carroll, J. S. FOXA1 is a key determinant of estrogen receptor function and endocrine response. *Nat. Genet.* **43**, 27–33 (2011). 21151129 10.1038/ng.730 PMC3024537

[10] Roe, J. S. et al. Enhancer reprogramming promotes pancreatic cancer metastasis. *Cell* **170**, 875–888 (2017). 28757253 10.1016/j.cell.2017.07.007 PMC5726277

[11] Chen, L. et al. A reinforcing HNF4–SMAD4 feed-forward module stabilizes enterocyte identity. *Nat. Genet.* **51**, 777–785 (2019). 30988513 10.1038/s41588-019-0384-0 PMC6650150

[12] Brunton, H., et al. HNF4A and GATA6 loss reveals therapeutically actionable subtypes in pancreatic cancer. *Cell Rep.* **31**, 107625 (2020). 32402285 10.1016/j.celrep.2020.107625 PMC9511995

[13] Okegawa, T., Ushio, K., Imai, M., Morimoto, M. & Hara, T. Orphan nuclear receptor HNF4G promotes bladder cancer growth and invasion through the regulation of the hyaluronan synthase 2 gene. *Oncogenesis* **2**, e58 (2013). 23896584 10.1038/oncsis.2013.25 PMC3740288

[14] Shukla, S. et al. Aberrant activation of a gastrointestinal transcriptional circuit in prostate cancer mediates castration resistance. *Cancer Cell* **32**, 792–806 (2017). 29153843 10.1016/j.ccell.2017.10.008 PMC5728174

[15] Wang, C. et al. Metformin inhibits pancreatic cancer metastasis caused by SMAD4 deficiency and consequent HNF4G upregulation. *Protein Cell* **12**, 128–144 (2021). 32737864 10.1007/s13238-020-00760-4 PMC7862466

[16] Wang, J. et al. Expression of HNF4G and its potential functions in lung cancer. *Oncotarget* **9**, 18018–18028 (2018). 29719587 10.18632/oncotarget.22933 PMC5915054

[17] Zhan, J., Zhang, Q., Tong, X., Liu, X. & Zhao, C. HNF4G stimulates the development of pancreatic cancer by promoting IGF2BP2 transcription. *Clin. Transl. Oncol.* **25**, 1472–1481 (2023). 36607591 10.1007/s12094-022-03048-7

[18] Klein, A. P. et al. Genome-wide meta-analysis identifies five new susceptibility loci for pancreatic cancer. *Nat. Commun.* **9**, 556 (2018). 29422604 10.1038/s41467-018-02942-5 PMC5805680

[19] Mohammed, H. & Carroll, J. S. Approaches for assessing and discovering protein interactions in cancer. *Mol. Cancer Res.* **11**, 1295–1302 (2013). 24072816 10.1158/1541-7786.MCR-13-0454 PMC3834224

[20] Mohammed, H. et al. Rapid immunoprecipitation mass spectrometry of endogenous proteins (RIME) for analysis of chromatin complexes. *Nat. Protoc.* **11**, 316–326 (2016). 26797456 10.1038/nprot.2016.020

[21] Papachristou, E. K. et al. A quantitative mass spectrometry-based approach to monitor the dynamics of endogenous chromatin-associated protein complexes. *Nat. Commun.* **9**, 2311 (2018). 29899353 10.1038/s41467-018-04619-5 PMC5998130

[22] Collisson, E. A. et al. Subtypes of pancreatic ductal adenocarcinoma and their differing responses to therapy. *Nat. Med.* **17**, 500 (2011). 21460848 10.1038/nm.2344 PMC3755490

[23] Martinelli, P. et al. GATA6 regulates EMT and tumour dissemination, and is a marker of response to adjuvant chemotherapy in pancreatic cancer. *Gut* **66**, 1665–1676 (2017). 27325420 10.1136/gutjnl-2015-311256 PMC5070637

[24] Chan-Seng-Yue, M. et al. Transcription phenotypes of pancreatic cancer are driven by genomic events during tumor evolution. *Nat. Genet.* **52**, 231–240 (2020). 31932696 10.1038/s41588-019-0566-9

[25] Isigkeit, L. & Merk, D. Opportunities and challenges in targeting orphan nuclear receptors. *Chem. Commun. (Camb.)* **59**, 4551–4561 (2023). 37000699 10.1039/d3cc00954h

[26] Wisely, G. B. et al. Hepatocyte nuclear factor 4 is a transcription factor that constitutively binds fatty acids. *Structure* **10**, 1225–1234 (2002). 12220494 10.1016/s0969-2126(02)00829-8

[27] Giuliani, V. et al. PRMT1-dependent regulation of RNA metabolism and DNA damage response sustains pancreatic ductal adenocarcinoma. *Nat. Commun.* **12**, 4626 (2021). 34330913 10.1038/s41467-021-24798-y PMC8324870

[28] Hingorani, S. R. et al. *Trp53*^*R172H*^ and *Kras*^*G12D*^ cooperate to promote chromosomal instability and widely metastatic pancreatic ductal adenocarcinoma in mice. *Cancer Cell* **7**, 469–483 (2005). 15894267 10.1016/j.ccr.2005.04.023

[29] Binker-Cosen, M. J. et al. Palmitic acid increases invasiveness of pancreatic cancer cells AsPC-1 through TLR4/ROS/NF-κB/MMP-9 signaling pathway. *Biochem. Biophys. Res. Commun.* **484**, 152–158 (2017). 28088520 10.1016/j.bbrc.2017.01.051

[30] Chen, L. et al. HNF4 regulates fatty acid oxidation and is required for renewal of intestinal stem cells in mice. *Gastroenterology* **158**, 985–999 (2020). 31759926 10.1053/j.gastro.2019.11.031 PMC7062567

[31] Kalisz, M. et al. HNF1A recruits KDM6A to activate differentiated acinar cell programs that suppress pancreatic cancer. *EMBO J.* **39**, e102808 (2020). 32154941 10.15252/embj.2019102808 PMC7196917

[32] Cirillo, L. A. & Zaret, K. S. An early developmental transcription factor complex that is more stable on nucleosome core particles than on free DNA. *Mol. Cell* **4**, 961–969 (1999). 10635321 10.1016/s1097-2765(00)80225-7

[33] Lupien, M. et al. FoxA1 translates epigenetic signatures into enhancer-driven lineage-specific transcription. *Cell* **132**, 958–970 (2008). 18358809 10.1016/j.cell.2008.01.018 PMC2323438

[34] Zhang, G. et al. FOXA1 defines cancer cell specificity. *Sci. Adv.* **2**, e1501473 (2016). 27034986 10.1126/sciadv.1501473 PMC4803482

[35] Ross-Innes, C. S. et al. Differential oestrogen receptor binding is associated with clinical outcome in breast cancer. *Nature* **481**, 389–393 (2012). 22217937 10.1038/nature10730 PMC3272464

[36] Broome, R. et al. TET2 is a component of the estrogen receptor complex and controls 5mC to 5hmC conversion at estrogen receptor *cis*-regulatory regions. *Cell Rep.* **34**, 108776 (2021). 33626359 10.1016/j.celrep.2021.108776 PMC7921846

[37] Jozwik, K. M., Chernukhin, I., Serandour, A. A., Nagarajan, S. & Carroll, J. S. FOXA1 directs H3K4 monomethylation at enhancers via recruitment of the methyltransferase MLL3. *Cell Rep.* **17**, 2715–2723 (2016). 27926873 10.1016/j.celrep.2016.11.028 PMC5177601

[38] Carroll, J. S. et al. Chromosome-wide mapping of estrogen receptor binding reveals long-range regulation requiring the forkhead protein FoxA1. *Cell* **122**, 33–43 (2005). 16009131 10.1016/j.cell.2005.05.008

[39] Mohammed, H. et al. Rapid immunoprecipitation mass spectrometry of endogenous proteins (RIME) for analysis of chromatin complexes. *Nat. Protoc.* **11**, 316 (2016). 26797456 10.1038/nprot.2016.020

[40] Tyanova, S. et al. The Perseus computational platform for comprehensive analysis of (prote)omics data. *Nat. Methods* **13**, 731–740 (2016). 27348712 10.1038/nmeth.3901

[41] Siersbæk, R. et al. IL6/STAT3 signaling hijacks estrogen receptor α enhancers to drive breast cancer metastasis. *Cancer Cell* **38**, 412–423 (2020). 32679107 10.1016/j.ccell.2020.06.007 PMC7116707

[42] Stark, R. & Brown, G. DiffBind: differential binding analysis of ChIP–seq peak data. bioconductor.org/packages/release/bioc/html/DiffBind.html (2011).

[43] Corces, M. R. et al. An improved ATAC-seq protocol reduces background and enables interrogation of frozen tissues. *Nat. Methods* **14**, 959–962 (2017). 28846090 10.1038/nmeth.4396 PMC5623106

[44] Lareau, C. A. & Aryee, M. J. Hichipper: a preprocessing pipeline for calling DNA loops from HiChIP data. *Nat. Methods* **15**, 155–156 (2018). 29489746 10.1038/nmeth.4583 PMC10572103

[45] Mumbach, M. R. et al. HiChIP: efficient and sensitive analysis of protein-directed genome architecture. *Nat. Methods* **13**, 919–922 (2016). 27643841 10.1038/nmeth.3999 PMC5501173

[46] Erstad, D. J. et al. Orthotopic and heterotopic murine models of pancreatic cancer and their different responses to FOLFIRINOX chemotherapy. *Dis. Model Mech.* **11**, dmm034793 (2018). 29903803 10.1242/dmm.034793 PMC6078400

[47] Couturier, D. L. et al. CarrollLab: transcription factor switching drives progression of the classical subtype of pancreatic cancer (v1.0). *Zenodo*10.5281/zenodo.16925967 (2025).

