# Supplementary material for: Transcription factor switching drives subtype-specific pancreatic cancer
Source: Nat Genet. 2025 Oct 30;57(12):3016–26. doi: 10.1038/s41588-025-02389-7 (PMC12695649; doi:10.1038/s41588-025-02389-7)
Supplement: Supplementary file 1 — Reporting Summary [file 41588_2025_2389_MOESM1_ESM.pdf]

nature portfolio

Corresponding author(s): Jason Carroll

Last updated by author(s): 2025/07/02

Reporting Summary

Nature Portfolio wishes to improve the reproducibility of the work that we publish. This form provides structure for consistency and transparency in reporting. For further information on Nature Portfolio policies, see our Editorial Policies and the Editorial Policy Checklist.

Statistics

For all statistical analyses, confirm that the following items are present in the figure legend, table legend, main text, or Methods section.

n/a	Confirmed	
☐	☒	The exact sample size (*n*) for each experimental group/condition, given as a discrete number and unit of measurement
☐	☒	A statement on whether measurements were taken from distinct samples or whether the same sample was measured repeatedly
☐	☒	The statistical test(s) used AND whether they are one- or two-sided<br>*Only common tests should be described solely by name; describe more complex techniques in the Methods section.*
☐	☒	A description of all covariates tested
☐	☒	A description of any assumptions or corrections, such as tests of normality and adjustment for multiple comparisons
☐	☒	A full description of the statistical parameters including central tendency (e.g. means) or other basic estimates (e.g. regression coefficient) AND variation (e.g. standard deviation) or associated estimates of uncertainty (e.g. confidence intervals)
☐	☐	For null hypothesis testing, the test statistic (e.g. *F*, *t*, *r*) with confidence intervals, effect sizes, degrees of freedom and *P* value noted<br>*Give P values as exact values whenever suitable.*
☒	☐	For Bayesian analysis, information on the choice of priors and Markov chain Monte Carlo settings
☐	☒	For hierarchical and complex designs, identification of the appropriate level for tests and full reporting of outcomes
☒	☐	Estimates of effect sizes (e.g. Cohen's *d*, Pearson's *r*), indicating how they were calculated

Our web collection on statistics for biologists contains articles on many of the points above.

Software and code

Policy information about availability of computer code

Data collection	No software's were used to generate the data.
Data analysis	bowtie2 v 2.2.6, DESeq2 v 1.30.1, qPLEXanalyzer tool (10.18129/B9.bioc.qPLEXanalyzer),MATLAB R2019b, MACS2 v 2.6.6, bedtools v 2.26.0-97, STAR version 2.6.1a, HiC-Pro 3.1.0, hichipper, version 0.7.9, Meme 4.9.1,5.0.5

For manuscripts utilizing custom algorithms or software that are central to the research but not yet described in published literature, software must be made available to editors and reviewers. We strongly encourage code deposition in a community repository (e.g. GitHub). See the Nature Portfolio guidelines for submitting code & software for further information.

Data

Policy information about availability of data

All manuscripts must include a data availability statement. This statement should provide the following information, where applicable:

- Accession codes, unique identifiers, or web links for publicly available datasets
- A description of any restrictions on data availability
- For clinical datasets or third party data, please ensure that the statement adheres to our policy

Data and Code Availability
All ChIP-seq, Hi-ChIP, ATAC-seq and RNA-seq data have been deposited at Gene Expression Omnibus and can be accessed at GSE245734.
All proteomic data have been deposited at PRIDE and can be accessed at PXD045980.

Source Data for Fig. 1 RIME is available online Fig. Table 1.
Source Data for RNA-seq is available online Fig. Table 2.

Research involving human participants, their data, or biological material

Policy information about studies with human participants or human data. See also policy information about sex, gender (identity/presentation), and sexual orientation and race, ethnicity and racism.

Reporting on sex and gender	NA
Reporting on race, ethnicity, or other socially relevant groupings	NA
Population characteristics	NA
Recruitment	NA
Ethics oversight	NA

Note that full information on the approval of the study protocol must also be provided in the manuscript.

Field-specific reporting

Please select the one below that is the best fit for your research. If you are not sure, read the appropriate sections before making your selection.

☒ Life sciences ☐ Behavioural & social sciences ☐ Ecological, evolutionary & environmental sciences

For a reference copy of the document with all sections, see nature.com/documents/nr-reporting-summary-flat.pdf

Life sciences study design

All studies must disclose on these points even when the disclosure is negative.

Sample size

In vivo orthotopic study of Figure 2:
this study considered 2 groups, Control and HNF4G-KO. The sample size per group (n=13, i.e., n=26 in total) was defined to detect a 30% reduction in tumour size at day 25 between the two groups with a probability (power) greater than 0.9 assuming [i] that tumour size per mouse and group as a function of time are normally distributed on the cube root scale with nuisance parameters estimated from data of a pilot experiment, [ii] the use of two-sided Wald t-test for the interaction parameters corresponding to the shift in slopes between groups in a random intercept and slope linear growth model [iii] a 5% type I error.

In vivo orthotopic study of Figure 4:
this study considered 4 groups, defined with combination of treatments (Control-Veh, Control-GSK) and mouse type (HNF4G-KO-Veh, HNF4G-KO-GSK) levels. The sample size per group (n=9 for the vehicle groups and n=15 for the treatment groups, i.e., n=48 in total) was defined so that the joint probability of detecting
• a median survival difference of 10 days between both vehicle groups,
• a median survival difference of 10 days between both HNF4G-KO groups,
• a median survival difference of 20 days between the groups 'Control-Veh' and 'HNF4-G-KO-GSK drug'
was greater than 80% (power) assuming [i] absence of censoring (full follow-up of all mice), [ii] survival times following a negative binomial distribution with nuisance parameters estimated from a previous study, [iii] the use of one-sided Mann-Whitney-Wilcoxon's test with 5% type I error, [iv] the use of unbalanced sample sizes per mouse type to optimise power.

In vivo Lung metastasis of Figure 4 (b &c)
This study involves four groups and was powered to detect pairwise differences in the average number of metastases between groups by means of a Poisson generalised linear model without multiplicity correction. Sample size calculation results showed that a sample size of n=10 mice per group would allow us to detect predefined differences of interest at the 5% level without multiplicity correction, even if the variance given the mean were to be inflated by 20% (when considering a Gamma mixture of Poisson, for example).

Data exclusions

NA

Replication

Na

Randomization

For both RNAseq and ChIPseq experiments, samples were block-randomised in a 96 well plate.

Mice were randomised into 4 groups (n=9 mice for Control Vehicle and HNF4G-KO Vehicle arms; n= 14 for Control + GSK3368715 arm and n=15 for HNF4G-KO + GSK3368715 arm). At 2 weeks post-surgery, mice were treated with either vehicle (Control, Vetivex saline) or GSK3368715 (75mg/kg) by oral gavage (5ml/kg), using a dosing regimen of 5 days on / 2 days off until clinical endpoint was reached.

Blinding

Ultra-sound volume measurements were done by two independent researchers completely blind to the cohorts of the drugs study.

Reporting for specific materials, systems and methods

We require information from authors about some types of materials, experimental systems and methods used in many studies. Here, indicate whether each material, system or method listed is relevant to your study. If you are not sure if a list item applies to your research, read the appropriate section before selecting a response.

Materials & experimental systems

n/a	Involved in the study
☐	☒ Antibodies
☐	☒ Eukaryotic cell lines
☐	☐ Palaeontology and archaeology
☐	☒ Animals and other organisms
☐	☐ Clinical data
☐	☐ Dual use research of concern
☐	☐ Plants

Methods

n/a	Involved in the study
☐	☒ ChIP-seq
☐	☐ Flow cytometry
☐	☐ MRI-based neuroimaging

Antibodies

Antibodies used: HNF4G (Atlas, HPA005438), HNF4A(Cell signalling, 3113S), PRMT1(Atlas, HPA072136), GATA6 (Cell signalling 5851, R&D systems AF1700) FOXA1 (abcam, ab5089), Mono-Methyl Arginine (MMA) #8015, Vinculin #13901 beta-actin #4970; Asymmetric Di-Methyl Arginine #13522, Sigma, A5441. Secondary antibodies: Goat anti-rabbit (926-32211, 926-68071), goat anti-mouse (926-32210, 926-68070), donkey anti-goat (926-32214).

Validation: Antibodies were validated with knockouts/knockdown experiments. Mass spectrometry-based approaches (RIME) and ChIP seq. Source data is provided in Table 1 for RIME experiments. GEO accessions numbers provided for all the ChIP seq data.

Eukaryotic cell lines

Policy information about cell lines and Sex and Gender in Research

Cell line source(s): AsPC-1, HPAF-II, BXPC-3, Panc-1 and MiaPaca (ATCC), KPC derived cell lines and CRISPR modified cell lines were generated at the CRUK, Cambridge Institute.

Authentication: Cells were routinely genotyped by short-tandem repeat (STR) genetic profiling using the Power Plex 16HS Cell Line panel and analyzed using the Applied Biosystems Gene Mapper ID v3.2.1 software by the external provider Genetica DNA Laboratories (LabCorp Specialty Testing Group).

Mycoplasma contamination: Cells were tested (MycoProbe Mycoplasma detection kit, R&D) around every major experiment and at least every six months. All cell lines were grown at 37°C.

Commonly misidentified lines (See ICLAC register): NA

Palaeontology and Archaeology

Specimen provenance: NA

Specimen deposition: NA

Dating methods: NA

☐ Tick this box to confirm that the raw and calibrated dates are available in the paper or in Supplementary Information.

Ethics oversight: NA

Note that full information on the approval of the study protocol must also be provided in the manuscript.

Animals and other research organisms

Policy information about studies involving animals; ARRIVE guidelines recommended for reporting animal research, and Sex and Gender in Research

Laboratory animals: All experiments were performed in accordance with the animal project licence (P40BD8F30) in accordance with home office guidelines.

	NOD scid gamma (NSG) mice were obtained from Charles River Laboratories, kept in pathogen-free conditions on a 12hr light-dark cycle and allowed to acclimatise for a period of 7 days before any surgery (at ~8 weeks old). Mice were grouped, housed with environment enrichment, fed on a maintenance diet (PicoLab) and housed with a room temperature of 22°C +/- 2°C and humidity of 55% +/- 10%.
Wild animals	NA
Reporting on sex	Wherever possible both genders were used in experimental procedures.
Field-collected samples	NA
Ethics oversight	NA

Note that full information on the approval of the study protocol must also be provided in the manuscript.

Clinical data

Policy information about clinical studies

All manuscripts should comply with the ICMJE guidelines for publication of clinical research and a completed CONSORT checklist must be included with all submissions.

Clinical trial registration	This study was approved by the East of England – Cambridgeshire and Hertfordshire REC Committee and is in compliance with GCP, local regulatory requirements and legal requirements for CAMPAN is 08/H0306/32. All patients provided written informed consent. All procedures performed in studies involving human participants were in accordance with the ethical standards of the institutional and/or national research committee and with the 1964 Helsinki declaration and its later amendments or comparable ethical standards. Release of data was also pseudo-anonymised as per the UK Human Tissue Act regulations.
Study protocol	Matched flash frozen and FFPE samples from Whipple Biopsies, undergoing surgical resection at the Cambridge University Hospitals were sectioned according to institutional protocols.<br>This study was approved by the East of England – Cambridgeshire and Hertfordshire REC Committee and is in compliance with GCP, local regulatory requirements and legal requirements for CAMPAN is 08/H0306/32. All patients provided written informed consent. All procedures performed in studies involving human participants were in accordance with the ethical standards of the institutional and/or national research committee and with the 1964 Helsinki declaration and its later amendments or comparable ethical standards. Release of data was also pseudo-anonymised as per the UK Human Tissue Act regulations.<br><br>Histological staining of FOXA1 and HNF4G in stage IV primary tumours and liver metastases was performed according to recommendations of the local ethics committee of the Medical Faculty of the University of Duisburg-Essen under approval No. 23-11451-B0. Clinical data were obtained from archives and electronic health records. All patients provided written informed consent.
Data collection	All data was anonymised. Data obtained from ChIP sequencing of the clinical samples has been deposited with GEO. Links to data has been provided.
Outcomes	NA

Dual use research of concern

Policy information about dual use research of concern

Hazards

Could the accidental, deliberate or reckless misuse of agents or technologies generated in the work, or the application of information presented in the manuscript, pose a threat to:

No	Yes	
☒	☐	Public health
☒	☐	National security
☒	☐	Crops and/or livestock
☒	☐	Ecosystems
☒	☐	Any other significant area

Experiments of concern

Does the work involve any of these experiments of concern:

No	Yes	
☒	☐	Demonstrate how to render a vaccine ineffective
☒	☐	Confer resistance to therapeutically useful antibiotics or antiviral agents
☒	☐	Enhance the virulence of a pathogen or render a nonpathogen virulent
☒	☐	Increase transmissibility of a pathogen
☒	☐	Alter the host range of a pathogen
☒	☐	Enable evasion of diagnostic/detection modalities
☒	☐	Enable the weaponization of a biological agent or toxin
☒	☐	Any other potentially harmful combination of experiments and agents

Plants

Seed stocks	NA
Novel plant genotypes	NA
Authentication	NA

ChIP-seq

Data deposition

☒ Confirm that both raw and final processed data have been deposited in a public database such as GEO.

☒ Confirm that you have deposited or provided access to graph files (e.g. BED files) for the called peaks.

Data access links
May remain private before publication.

https://www.ncbi.nlm.nih.gov/geo/query/acc.cgi?acc=GSE245732

Files in database submission

hpaf17_HPAFII_PRMT1_Cas_9_CRI02_1.fq.gz
hpaf17_HPAFII_PRMT1_Cas_9_CRI02_2.fq.gz
hpaf18_HPAFII_PRMT1_Cas_9_CRI02_1.fq.gz
hpaf18_HPAFII_PRMT1_Cas_9_CRI02_2.fq.gz
hpaf19_HPAFII_PRMT1_Cas_9_CRI02_1.fq.gz
hpaf19_HPAFII_PRMT1_Cas_9_CRI02_2.fq.gz
hpaf1_HPAFII_FOXA1_Cas_9_CRI02_1.fq.gz
hpaf1_HPAFII_FOXA1_Cas_9_CRI02_2.fq.gz
hpaf20_HPAFII_PRMT1_HNF4G_KO_CRI02_1.fq.gz
hpaf20_HPAFII_PRMT1_HNF4G_KO_CRI02_2.fq.gz
hpaf21_HPAFII_PRMT1_HNF4G_KO_CRI02_1.fq.gz
hpaf21_HPAFII_PRMT1_HNF4G_KO_CRI02_2.fq.gz
hpaf22_HPAFII_PRMT1_HNF4G_KO_CRI02_1.fq.gz
hpaf22_HPAFII_PRMT1_HNF4G_KO_CRI02_2.fq.gz
hpaf23_HPAFII_HNF4G_Non_targetting_siRNA_CRI02_1.fq.gz
hpaf23_HPAFII_HNF4G_Non_targetting_siRNA_CRI02_2.fq.gz
hpaf24_HPAFII_HNF4G_FOXA1_sirna_CRI02_1.fq.gz
hpaf24_HPAFII_HNF4G_FOXA1_sirna_CRI02_2.fq.gz
hpaf25_HPAFII_HNF4G_Non_targetting_siRNA_CRI02_1.fq.gz
hpaf25_HPAFII_HNF4G_Non_targetting_siRNA_CRI02_2.fq.gz
hpaf26_HPAFII_HNF4G_FOXA1_sirna_CRI02_1.fq.gz
hpaf26_HPAFII_HNF4G_FOXA1_sirna_CRI02_2.fq.gz
hpaf27_HPAFII_HNF4G_Non_targetting_siRNA_CRI02_1.fq.gz
hpaf27_HPAFII_HNF4G_Non_targetting_siRNA_CRI02_2.fq.gz
hpaf28_HPAFII_HNF4G_FOXA1_sirna_CRI02_1.fq.gz
hpaf28_HPAFII_HNF4G_FOXA1_sirna_CRI02_2.fq.gz
hpaf30_HPAFII_HNF4G_FOXA1_sirna_CRI02_1.fq.gz
hpaf30_HPAFII_HNF4G_FOXA1_sirna_CRI02_2.fq.gz
hpaf31_HPAFII_H3K27Ac_Cas_9_CRI02_1.fq.gz

hpaf31_HPAFII_H3K27Ac_Cas_9_CRI02_2.fq.gz
hpaf32_HPAFII_H3K27Ac_Cas_9_CRI02_1.fq.gz
hpaf32_HPAFII_H3K27Ac_Cas_9_CRI02_2.fq.gz
hpaf33_HPAFII_H3K27Ac_Cas_9_CRI02_1.fq.gz
hpaf33_HPAFII_H3K27Ac_Cas_9_CRI02_2.fq.gz
hpaf34_HPAFII_H3K27Ac_Cas_9_CRI02_1.fq.gz
hpaf34_HPAFII_H3K27Ac_Cas_9_CRI02_2.fq.gz
hpaf35_HPAFII_H3K27Ac_HNF4G_KO_CRI02_1.fq.gz
hpaf35_HPAFII_H3K27Ac_HNF4G_KO_CRI02_2.fq.gz
hpaf36_HPAFII_H3K27Ac_HNF4G_KO_CRI02_1.fq.gz
hpaf36_HPAFII_H3K27Ac_HNF4G_KO_CRI02_2.fq.gz
hpaf37_HPAFII_H3K27Ac_HNF4G_KO_CRI02_1.fq.gz
hpaf37_HPAFII_H3K27Ac_HNF4G_KO_CRI02_2.fq.gz
hpaf39_HPAFII_H3K27Ac_Non_targetting_siRNA_CRI02_1.fq.gz
hpaf39_HPAFII_H3K27Ac_Non_targetting_siRNA_CRI02_2.fq.gz
hpaf3_HPAFII_FOXA1_Cas_9_CRI02_1.fq.gz
hpaf3_HPAFII_FOXA1_Cas_9_CRI02_2.fq.gz
hpaf40_HPAFII_H3K27Ac_FOXA1_sirna_CRI02_1.fq.gz
hpaf40_HPAFII_H3K27Ac_FOXA1_sirna_CRI02_2.fq.gz
hpaf41_HPAFII_H3K27Ac_Non_targetting_siRNA_CRI02_1.fq.gz
hpaf41_HPAFII_H3K27Ac_Non_targetting_siRNA_CRI02_2.fq.gz
hpaf42_HPAFII_H3K27Ac_FOXA1_sirna_CRI02_1.fq.gz
hpaf42_HPAFII_H3K27Ac_FOXA1_sirna_CRI02_2.fq.gz
hpaf43_HPAFII_H3K27Ac_Non_targetting_siRNA_CRI02_1.fq.gz
hpaf43_HPAFII_H3K27Ac_Non_targetting_siRNA_CRI02_2.fq.gz
hpaf44_HPAFII_H3K27Ac_FOXA1_sirna_CRI02_1.fq.gz
hpaf44_HPAFII_H3K27Ac_FOXA1_sirna_CRI02_2.fq.gz
hpaf45_HPAFII_H3K27Ac_Non_targetting_siRNA_CRI02_1.fq.gz
hpaf45_HPAFII_H3K27Ac_Non_targetting_siRNA_CRI02_2.fq.gz
hpaf46_HPAFII_H3K27Ac_FOXA1_sirna_CRI02_1.fq.gz
hpaf46_HPAFII_H3K27Ac_FOXA1_sirna_CRI02_2.fq.gz
hpaf47_HPAFII_Input_Inputs_CRI02_1.fq.gz
hpaf47_HPAFII_Input_Inputs_CRI02_2.fq.gz
hpaf48_HPAFII_Input_Inputs_CRI02_1.fq.gz
hpaf48_HPAFII_Input_Inputs_CRI02_2.fq.gz
hpaf49_HPAFII_Input_Inputs_CRI02_1.fq.gz
hpaf49_HPAFII_Input_Inputs_CRI02_2.fq.gz
hpaf4_HPAFII_FOXA1_Cas_9_CRI02_1.fq.gz
hpaf4_HPAFII_FOXA1_Cas_9_CRI02_2.fq.gz
hpaf50_HPAFII_Input_Inputs_CRI02_1.fq.gz
hpaf50_HPAFII_Input_Inputs_CRI02_2.fq.gz
hpaf6_HPAFII_FOXA1_HNF4G_KO_CRI02_1.fq.gz
hpaf6_HPAFII_FOXA1_HNF4G_KO_CRI02_2.fq.gz
hpaf7_HPAFII_FOXA1_HNF4G_KO_CRI02_1.fq.gz
hpaf7_HPAFII_FOXA1_HNF4G_KO_CRI02_2.fq.gz
hpaf8_HPAFII_FOXA1_HNF4G_KO_CRI02_1.fq.gz
hpaf8_HPAFII_FOXA1_HNF4G_KO_CRI02_2.fq.gz
jc6495_KPC_METS_FOXA1_CRI08.fq.gz
jc6614_KPC_tumour_FOXA1_untreated_CRI02_1.fq.gz
jc6614_KPC_tumour_FOXA1_untreated_CRI02_2.fq.gz
jc6632_KPC_tumour_HNF4G_untreated_CRI02_1.fq.gz
jc6632_KPC_tumour_HNF4G_untreated_CRI02_2.fq.gz
jc6633_KPC_mets_HNF4G_untreated_CRI02_1.fq.gz
jc6633_KPC_mets_HNF4G_untreated_CRI02_2.fq.gz
jc6640_KPC_tumour_input_untreated_CRI02_1.fq.gz
jc6640_KPC_tumour_input_untreated_CRI02_2.fq.gz
jc6641_KPC_mets_input_untreated_CRI02_1.fq.gz
jc6641_KPC_mets_input_untreated_CRI02_2.fq.gz
jc6725_AsPC_1_HNF4G_CRI02_1.fq.gz
jc6725_AsPC_1_HNF4G_CRI02_2.fq.gz
jc6726_AsPC_1_HNF4G_CRI02_1.fq.gz
jc6726_AsPC_1_HNF4G_CRI02_2.fq.gz
jc6729_AsPC_1_FOXA1_CRI02_1.fq.gz
jc6729_AsPC_1_FOXA1_CRI02_2.fq.gz
jc6730_AsPC_1_FOXA1_CRI02_1.fq.gz
jc6730_AsPC_1_FOXA1_CRI02_2.fq.gz
jc6731_AsPC_1_HNF4A_CRI02_1.fq.gz
jc6731_AsPC_1_HNF4A_CRI02_2.fq.gz
jc6733_AsPC_1_HNF4A_CRI02_1.fq.gz
jc6733_AsPC_1_HNF4A_CRI02_2.fq.gz
jc6735_AsPC_1_GATA6_CRI02_1.fq.gz
jc6735_AsPC_1_GATA6_CRI02_2.fq.gz
jc7464_jc7464_FOXA1_CRI01_1.fq.gz
jc7464_jc7464_FOXA1_CRI01_2.fq.gz
jc7465_jc7465_HNF4a_CRI01_1.fq.gz
jc7465_jc7465_HNF4a_CRI01_2.fq.gz

jc7466_jc7466_HNF4g_CRI01_1.fq.gz
jc7466_jc7466_HNF4g_CRI01_2.fq.gz
jc7467_jc7467_FOXA1_CRI01_1.fq.gz
jc7467_jc7467_FOXA1_CRI01_2.fq.gz
jc7468_jc7468_HNF4a_CRI01_1.fq.gz
jc7468_jc7468_HNF4a_CRI01_2.fq.gz
jc7469_jc7469_HNF4g_CRI01_1.fq.gz
jc7469_jc7469_HNF4g_CRI01_2.fq.gz
jc7470_jc7470_FOXA1_CRI01_1.fq.gz
jc7470_jc7470_FOXA1_CRI01_2.fq.gz
jc7471_jc7471_HNF4a_CRI01_1.fq.gz
jc7471_jc7471_HNF4a_CRI01_2.fq.gz
jc7472_jc7472_HNF4g_CRI01_1.fq.gz
jc7472_jc7472_HNF4g_CRI01_2.fq.gz
jc7473_jc7473_FOXA1_CRI01_1.fq.gz
jc7473_jc7473_FOXA1_CRI01_2.fq.gz
jc7474_jc7474_HNF4a_CRI01_1.fq.gz
jc7474_jc7474_HNF4a_CRI01_2.fq.gz
jc7475_jc7475_HNF4g_CRI01_1.fq.gz
jc7475_jc7475_HNF4g_CRI01_2.fq.gz
jc7476_jc7476_FOXA1_CRI01_1.fq.gz
jc7476_jc7476_FOXA1_CRI01_2.fq.gz
jc7477_jc7477_HNF4a_CRI01_1.fq.gz
jc7477_jc7477_HNF4a_CRI01_2.fq.gz
jc7478_jc7478_HNF4g_CRI01_1.fq.gz
jc7478_jc7478_HNF4g_CRI01_2.fq.gz
jc7479_jc7479_FOXA1_CRI01_1.fq.gz
jc7479_jc7479_FOXA1_CRI01_2.fq.gz
jc7480_jc7480_HNF4a_CRI01_1.fq.gz
jc7480_jc7480_HNF4a_CRI01_2.fq.gz
jc7481_jc7481_HNF4g_CRI01_1.fq.gz
jc7481_jc7481_HNF4g_CRI01_2.fq.gz
jc7482_jc7482_INPUT_CRI01_1.fq.gz
jc7482_jc7482_INPUT_CRI01_2.fq.gz
jc7483_jc7483_INPUT_CRI01_1.fq.gz
jc7483_jc7483_INPUT_CRI01_2.fq.gz
jc7484_jc7484_INPUT_CRI01_1.fq.gz
jc7484_jc7484_INPUT_CRI01_2.fq.gz
jc7485_jc7485_INPUT_CRI01_1.fq.gz
jc7485_jc7485_INPUT_CRI01_2.fq.gz
jc7486_jc7486_INPUT_CRI01_1.fq.gz
jc7486_jc7486_INPUT_CRI01_2.fq.gz
jc7487_jc7487_INPUT_CRI01_1.fq.gz
jc7487_jc7487_INPUT_CRI01_2.fq.gz
jc7488_jc7488_H3K27Ac_CRI02_1.fq.gz
jc7488_jc7488_H3K27Ac_CRI02_2.fq.gz
jc7489_jc7489_H3K27Ac_CRI02_1.fq.gz
jc7489_jc7489_H3K27Ac_CRI02_2.fq.gz
jc7490_jc7490_H3K27Ac_CRI02_1.fq.gz
jc7490_jc7490_H3K27Ac_CRI02_2.fq.gz
jc7491_jc7491_H3K27Ac_CRI02_1.fq.gz
jc7491_jc7491_H3K27Ac_CRI02_2.fq.gz
jc7492_jc7492_H3K27Ac_CRI02_1.fq.gz
jc7492_jc7492_H3K27Ac_CRI02_2.fq.gz
jc7493_jc7493_INPUT_CRI02_1.fq.gz
jc7493_jc7493_INPUT_CRI02_2.fq.gz
jc7494_jc7494_INPUT_CRI02_1.fq.gz
jc7494_jc7494_INPUT_CRI02_2.fq.gz
jc7495_jc7495_INPUT_CRI02_1.fq.gz
jc7495_jc7495_INPUT_CRI02_2.fq.gz
jc7496_jc7496_INPUT_CRI02_1.fq.gz
jc7496_jc7496_INPUT_CRI02_2.fq.gz
jc7497_jc7497_INPUT_CRI02_1.fq.gz
jc7497_jc7497_INPUT_CRI02_2.fq.gz
jc7832_jc7832_H3K27Ac_Tumour_CRI01_1.fq.gz
jc7832_jc7832_H3K27Ac_Tumour_CRI01_2.fq.gz
jc7833_jc7833_H3K27Ac_Tumour_CRI01_1.fq.gz
jc7833_jc7833_H3K27Ac_Tumour_CRI01_2.fq.gz
jc7834_jc7834_H3K27Ac_Tumour_CRI01_1.fq.gz
jc7834_jc7834_H3K27Ac_Tumour_CRI01_2.fq.gz
jc7835_jc7835_H3K27Ac_Tumour_CRI01_1.fq.gz
jc7835_jc7835_H3K27Ac_Tumour_CRI01_2.fq.gz
jc7836_jc7836_H3K27Ac_Tumour_CRI01_1.fq.gz
jc7836_jc7836_H3K27Ac_Tumour_CRI01_2.fq.gz
jc7837_jc7837_H3K27Ac_normal_CRI01_1.fq.gz
jc7837_jc7837_H3K27Ac_normal_CRI01_2.fq.gz

jc7838_jc7838_H3K27Ac_normal_CRI01_1.fq.gz
jc7838_jc7838_H3K27Ac_normal_CRI01_2.fq.gz
jc7839_jc7839_H3K27Ac_normal_CRI01_1.fq.gz
jc7839_jc7839_H3K27Ac_normal_CRI01_2.fq.gz
jc7840_jc7840_H3K27Ac_normal_CRI01_1.fq.gz
jc7840_jc7840_H3K27Ac_normal_CRI01_2.fq.gz
jc7841_jc7841_H3K27Ac_Tumour_CRI01_1.fq.gz
jc7841_jc7841_H3K27Ac_Tumour_CRI01_2.fq.gz
jc7842_jc7842_INPUT_Tumour_CRI01_1.fq.gz
jc7842_jc7842_INPUT_Tumour_CRI01_2.fq.gz
jc7843_jc7843_INPUT_Tumour_CRI01_1.fq.gz
jc7843_jc7843_INPUT_Tumour_CRI01_2.fq.gz
jc7844_jc7844_INPUT_Tumour_CRI01_1.fq.gz
jc7844_jc7844_INPUT_Tumour_CRI01_2.fq.gz
jc7845_jc7845_INPUT_Tumour_CRI01_1.fq.gz
jc7845_jc7845_INPUT_Tumour_CRI01_2.fq.gz
jc7846_jc7846_INPUT_Tumour_CRI01_1.fq.gz
jc7846_jc7846_INPUT_Tumour_CRI01_2.fq.gz
jc7847_jc7847_INPUT_Tumour_CRI01_1.fq.gz
jc7847_jc7847_INPUT_Tumour_CRI01_2.fq.gz
jc7848_jc7848_INPUT_normal_CRI01_1.fq.gz
jc7848_jc7848_INPUT_normal_CRI01_2.fq.gz
jc7849_jc7849_INPUT_normal_CRI01_1.fq.gz
jc7849_jc7849_INPUT_normal_CRI01_2.fq.gz
jc7850_jc7850_INPUT_normal_CRI01_1.fq.gz
jc7850_jc7850_INPUT_normal_CRI01_2.fq.gz
jc7851_jc7851_INPUT_normal_CRI01_1.fq.gz
jc7851_jc7851_INPUT_normal_CRI01_2.fq.gz
jc7852_jc7852_INPUT_Tumour_CRI01_1.fq.gz
jc7852_jc7852_INPUT_Tumour_CRI01_2.fq.gz
jc8009_jc8009_PRMT1_Tumour_CRI02_1.fq.gz
jc8009_jc8009_PRMT1_Tumour_CRI02_2.fq.gz
jc8010_jc8010_PRMT1_Tumour_CRI02_1.fq.gz
jc8010_jc8010_PRMT1_Tumour_CRI02_2.fq.gz
jc8011_jc8011_PRMT1_Tumour_CRI02_1.fq.gz
jc8011_jc8011_PRMT1_Tumour_CRI02_2.fq.gz
jc8012_jc8012_PRMT1_Tumour_CRI02_1.fq.gz
jc8012_jc8012_PRMT1_Tumour_CRI02_2.fq.gz
jc8013_jc8013_PRMT1_Tumour_CRI02_1.fq.gz
jc8013_jc8013_PRMT1_Tumour_CRI02_2.fq.gz
jc8014_jc8014_PRMT1_Tumour_CRI02_1.fq.gz
jc8014_jc8014_PRMT1_Tumour_CRI02_2.fq.gz
jc8135_HPAFII_PRMT1_Cas_9_CRI02_1.fq.gz
jc8135_HPAFII_PRMT1_Cas_9_CRI02_2.fq.gz
jc8136_HPAFII_PRMT1_Cas_9_CRI02_1.fq.gz
jc8136_HPAFII_PRMT1_Cas_9_CRI02_2.fq.gz
jc8137_HPAFII_PRMT1_Cas_9_CRI02_1.fq.gz
jc8137_HPAFII_PRMT1_Cas_9_CRI02_2.fq.gz
jc8138_HPAFII_PRMT1_HNF4G_KO_Rescue_CRI02_1.fq.gz
jc8138_HPAFII_PRMT1_HNF4G_KO_Rescue_CRI02_2.fq.gz
jc8139_HPAFII_PRMT1_HNF4G_KO_Rescue_CRI02_1.fq.gz
jc8139_HPAFII_PRMT1_HNF4G_KO_Rescue_CRI02_2.fq.gz
jc8140_HPAFII_PRMT1_HNF4G_KO_Rescue_CRI02_1.fq.gz
jc8140_HPAFII_PRMT1_HNF4G_KO_Rescue_CRI02_2.fq.gz
jc8142_HPAFII_PRMT1_Cas_9_NT_CRI02_1.fq.gz
jc8142_HPAFII_PRMT1_Cas_9_NT_CRI02_2.fq.gz
jc8143_HPAFII_PRMT1_Cas_9_NT_CRI02_1.fq.gz
jc8143_HPAFII_PRMT1_Cas_9_NT_CRI02_2.fq.gz
jc8144_HPAFII_PRMT1_Cas_9_NT_CRI02_1.fq.gz
jc8144_HPAFII_PRMT1_Cas_9_NT_CRI02_2.fq.gz
jc8145_HPAFII_PRMT1_Cas_9_FOXA1_siRNA_CRI02_1.fq.gz
jc8145_HPAFII_PRMT1_Cas_9_FOXA1_siRNA_CRI02_2.fq.gz
jc8146_HPAFII_PRMT1_Cas_9_FOXA1_siRNA_CRI02_1.fq.gz
jc8146_HPAFII_PRMT1_Cas_9_FOXA1_siRNA_CRI02_2.fq.gz
jc8147_HPAFII_PRMT1_Cas_9_FOXA1_siRNA_CRI02_1.fq.gz
jc8147_HPAFII_PRMT1_Cas_9_FOXA1_siRNA_CRI02_2.fq.gz
jc8148_HPAFII_PRMT1_Input_Cas_9_EV_CRI02_1.fq.gz
jc8148_HPAFII_PRMT1_Input_Cas_9_EV_CRI02_2.fq.gz
jc8149_HPAFII_PRMT1_Input_HNF4G_KO_Rescue_CRI02_1.fq.gz
jc8149_HPAFII_PRMT1_Input_HNF4G_KO_Rescue_CRI02_2.fq.gz
jc8150_HPAFII_PRMT1_Input_HNF4G_KO_CRI02_1.fq.gz
jc8150_HPAFII_PRMT1_Input_HNF4G_KO_CRI02_2.fq.gz
jc8363_HPAFII_FOXA1_Cas_9_EV_CRI01_1.fq.gz
jc8363_HPAFII_FOXA1_Cas_9_EV_CRI01_2.fq.gz
jc8363_HPAFII_FOXA1_Cas_9_EV_CRI02_1.fq.gz
jc8363_HPAFII_FOXA1_Cas_9_EV_CRI02_2.fq.gz

jc8363_HPAFII_FOXA1_Cas_9_EV_CRI11_1.fq.gz
jc8363_HPAFII_FOXA1_Cas_9_EV_CRI11_2.fq.gz
jc8363_HPAFII_FOXA1_Cas_9_EV_CRI12_1.fq.gz
jc8363_HPAFII_FOXA1_Cas_9_EV_CRI12_2.fq.gz
jc8364_HPAFII_FOXA1_Cas_9_FOXA1_CRI01_1.fq.gz
jc8364_HPAFII_FOXA1_Cas_9_FOXA1_CRI01_2.fq.gz
jc8364_HPAFII_FOXA1_Cas_9_FOXA1_CRI02_1.fq.gz
jc8364_HPAFII_FOXA1_Cas_9_FOXA1_CRI02_2.fq.gz
jc8364_HPAFII_FOXA1_Cas_9_FOXA1_CRI11_1.fq.gz
jc8364_HPAFII_FOXA1_Cas_9_FOXA1_CRI11_2.fq.gz
jc8364_HPAFII_FOXA1_Cas_9_FOXA1_CRI12_1.fq.gz
jc8364_HPAFII_FOXA1_Cas_9_FOXA1_CRI12_2.fq.gz
jc8365_HPAFII_FOXA1_HNF4G_KO_FOXA1_CRI01_1.fq.gz
jc8365_HPAFII_FOXA1_HNF4G_KO_FOXA1_CRI01_2.fq.gz
jc8365_HPAFII_FOXA1_HNF4G_KO_FOXA1_CRI02_1.fq.gz
jc8365_HPAFII_FOXA1_HNF4G_KO_FOXA1_CRI02_2.fq.gz
jc8365_HPAFII_FOXA1_HNF4G_KO_FOXA1_CRI11_1.fq.gz
jc8365_HPAFII_FOXA1_HNF4G_KO_FOXA1_CRI11_2.fq.gz
jc8365_HPAFII_FOXA1_HNF4G_KO_FOXA1_CRI12_1.fq.gz
jc8365_HPAFII_FOXA1_HNF4G_KO_FOXA1_CRI12_2.fq.gz
jc8366_HPAFII_FOXA1_Cas_9_EV_CRI01_1.fq.gz
jc8366_HPAFII_FOXA1_Cas_9_EV_CRI01_2.fq.gz
jc8366_HPAFII_FOXA1_Cas_9_EV_CRI02_1.fq.gz
jc8366_HPAFII_FOXA1_Cas_9_EV_CRI02_2.fq.gz
jc8366_HPAFII_FOXA1_Cas_9_EV_CRI11_1.fq.gz
jc8366_HPAFII_FOXA1_Cas_9_EV_CRI11_2.fq.gz
jc8366_HPAFII_FOXA1_Cas_9_EV_CRI12_1.fq.gz
jc8366_HPAFII_FOXA1_Cas_9_EV_CRI12_2.fq.gz
jc8367_HPAFII_FOXA1_Cas_9_FOXA1_CRI01_1.fq.gz
jc8367_HPAFII_FOXA1_Cas_9_FOXA1_CRI01_2.fq.gz
jc8367_HPAFII_FOXA1_Cas_9_FOXA1_CRI02_1.fq.gz
jc8367_HPAFII_FOXA1_Cas_9_FOXA1_CRI02_2.fq.gz
jc8367_HPAFII_FOXA1_Cas_9_FOXA1_CRI11_1.fq.gz
jc8367_HPAFII_FOXA1_Cas_9_FOXA1_CRI11_2.fq.gz
jc8367_HPAFII_FOXA1_Cas_9_FOXA1_CRI12_1.fq.gz
jc8367_HPAFII_FOXA1_Cas_9_FOXA1_CRI12_2.fq.gz
jc8368_HPAFII_FOXA1_HNF4G_KO_FOXA1_CRI01_1.fq.gz
jc8368_HPAFII_FOXA1_HNF4G_KO_FOXA1_CRI01_2.fq.gz
jc8368_HPAFII_FOXA1_HNF4G_KO_FOXA1_CRI02_1.fq.gz
jc8368_HPAFII_FOXA1_HNF4G_KO_FOXA1_CRI02_2.fq.gz
jc8368_HPAFII_FOXA1_HNF4G_KO_FOXA1_CRI11_1.fq.gz
jc8368_HPAFII_FOXA1_HNF4G_KO_FOXA1_CRI11_2.fq.gz
jc8368_HPAFII_FOXA1_HNF4G_KO_FOXA1_CRI12_1.fq.gz
jc8368_HPAFII_FOXA1_HNF4G_KO_FOXA1_CRI12_2.fq.gz
jc8370_HPAFII_FOXA1_Cas_9_FOXA1_CRI01_1.fq.gz
jc8370_HPAFII_FOXA1_Cas_9_FOXA1_CRI01_2.fq.gz
jc8370_HPAFII_FOXA1_Cas_9_FOXA1_CRI02_1.fq.gz
jc8370_HPAFII_FOXA1_Cas_9_FOXA1_CRI02_2.fq.gz
jc8371_HPAFII_FOXA1_HNF4G_KO_FOXA1_CRI01_1.fq.gz
jc8371_HPAFII_FOXA1_HNF4G_KO_FOXA1_CRI01_2.fq.gz
jc8371_HPAFII_FOXA1_HNF4G_KO_FOXA1_CRI02_1.fq.gz
jc8371_HPAFII_FOXA1_HNF4G_KO_FOXA1_CRI02_2.fq.gz
jc8371_HPAFII_FOXA1_HNF4G_KO_FOXA1_CRI11_1.fq.gz
jc8371_HPAFII_FOXA1_HNF4G_KO_FOXA1_CRI11_2.fq.gz
jc8371_HPAFII_FOXA1_HNF4G_KO_FOXA1_CRI12_1.fq.gz
jc8371_HPAFII_FOXA1_HNF4G_KO_FOXA1_CRI12_2.fq.gz
jc8372_HPAFII_FOXA1_Cas_9_EV_CRI01_1.fq.gz
jc8372_HPAFII_FOXA1_Cas_9_EV_CRI01_2.fq.gz
jc8372_HPAFII_FOXA1_Cas_9_EV_CRI02_1.fq.gz
jc8372_HPAFII_FOXA1_Cas_9_EV_CRI02_2.fq.gz
jc8372_HPAFII_FOXA1_Cas_9_EV_CRI11_1.fq.gz
jc8372_HPAFII_FOXA1_Cas_9_EV_CRI11_2.fq.gz
jc8372_HPAFII_FOXA1_Cas_9_EV_CRI12_1.fq.gz
jc8372_HPAFII_FOXA1_Cas_9_EV_CRI12_2.fq.gz
jc8373_HPAFII_FOXA1_Cas_9_FOXA1_CRI01_1.fq.gz
jc8373_HPAFII_FOXA1_Cas_9_FOXA1_CRI01_2.fq.gz
jc8373_HPAFII_FOXA1_Cas_9_FOXA1_CRI02_1.fq.gz
jc8373_HPAFII_FOXA1_Cas_9_FOXA1_CRI02_2.fq.gz
jc8373_HPAFII_FOXA1_Cas_9_FOXA1_CRI11_1.fq.gz
jc8373_HPAFII_FOXA1_Cas_9_FOXA1_CRI11_2.fq.gz
jc8373_HPAFII_FOXA1_Cas_9_FOXA1_CRI12_1.fq.gz
jc8373_HPAFII_FOXA1_Cas_9_FOXA1_CRI12_2.fq.gz
jc8376_HPAFII_HNF4G_Cas_9_FOXA1_CRI01_1.fq.gz
jc8376_HPAFII_HNF4G_Cas_9_FOXA1_CRI01_2.fq.gz
jc8376_HPAFII_HNF4G_Cas_9_FOXA1_CRI02_1.fq.gz
jc8376_HPAFII_HNF4G_Cas_9_FOXA1_CRI02_2.fq.gz

jc8376_HPAFII_HNF4G_Cas_9_FOXA1_CRI11_1.fq.gz
jc8376_HPAFII_HNF4G_Cas_9_FOXA1_CRI11_2.fq.gz
jc8376_HPAFII_HNF4G_Cas_9_FOXA1_CRI12_1.fq.gz
jc8376_HPAFII_HNF4G_Cas_9_FOXA1_CRI12_2.fq.gz
jc8377_HPAFII_HNF4G_HNF4G_KO_FOXA1_CRI01_1.fq.gz
jc8377_HPAFII_HNF4G_HNF4G_KO_FOXA1_CRI01_2.fq.gz
jc8377_HPAFII_HNF4G_HNF4G_KO_FOXA1_CRI02_1.fq.gz
jc8377_HPAFII_HNF4G_HNF4G_KO_FOXA1_CRI02_2.fq.gz
jc8377_HPAFII_HNF4G_HNF4G_KO_FOXA1_CRI11_1.fq.gz
jc8377_HPAFII_HNF4G_HNF4G_KO_FOXA1_CRI11_2.fq.gz
jc8377_HPAFII_HNF4G_HNF4G_KO_FOXA1_CRI12_1.fq.gz
jc8377_HPAFII_HNF4G_HNF4G_KO_FOXA1_CRI12_2.fq.gz
jc8378_HPAFII_HNF4G_Cas_9_EV_CRI01_1.fq.gz
jc8378_HPAFII_HNF4G_Cas_9_EV_CRI01_2.fq.gz
jc8378_HPAFII_HNF4G_Cas_9_EV_CRI02_1.fq.gz
jc8378_HPAFII_HNF4G_Cas_9_EV_CRI02_2.fq.gz
jc8379_HPAFII_HNF4G_Cas_9_FOXA1_CRI01_1.fq.gz
jc8379_HPAFII_HNF4G_Cas_9_FOXA1_CRI01_2.fq.gz
jc8379_HPAFII_HNF4G_Cas_9_FOXA1_CRI02_1.fq.gz
jc8379_HPAFII_HNF4G_Cas_9_FOXA1_CRI02_2.fq.gz
jc8379_HPAFII_HNF4G_Cas_9_FOXA1_CRI11_1.fq.gz
jc8379_HPAFII_HNF4G_Cas_9_FOXA1_CRI11_2.fq.gz
jc8379_HPAFII_HNF4G_Cas_9_FOXA1_CRI12_1.fq.gz
jc8379_HPAFII_HNF4G_Cas_9_FOXA1_CRI12_2.fq.gz
jc8380_HPAFII_HNF4G_Cas_9_EV_CRI01_1.fq.gz
jc8380_HPAFII_HNF4G_Cas_9_EV_CRI01_2.fq.gz
jc8380_HPAFII_HNF4G_Cas_9_EV_CRI02_1.fq.gz
jc8380_HPAFII_HNF4G_Cas_9_EV_CRI02_2.fq.gz
jc8380_HPAFII_HNF4G_Cas_9_EV_CRI11_1.fq.gz
jc8380_HPAFII_HNF4G_Cas_9_EV_CRI11_2.fq.gz
jc8380_HPAFII_HNF4G_Cas_9_EV_CRI12_1.fq.gz
jc8380_HPAFII_HNF4G_Cas_9_EV_CRI12_2.fq.gz
jc8381_HPAFII_HNF4G_Cas_9_FOXA1_CRI01_1.fq.gz
jc8381_HPAFII_HNF4G_Cas_9_FOXA1_CRI01_2.fq.gz
jc8381_HPAFII_HNF4G_Cas_9_FOXA1_CRI02_1.fq.gz
jc8381_HPAFII_HNF4G_Cas_9_FOXA1_CRI02_2.fq.gz
jc8381_HPAFII_HNF4G_Cas_9_FOXA1_CRI11_1.fq.gz
jc8381_HPAFII_HNF4G_Cas_9_FOXA1_CRI11_2.fq.gz
jc8381_HPAFII_HNF4G_Cas_9_FOXA1_CRI12_1.fq.gz
jc8381_HPAFII_HNF4G_Cas_9_FOXA1_CRI12_2.fq.gz
jc8382_HPAFII_HNF4G_Cas_9_EV_CRI01_1.fq.gz
jc8382_HPAFII_HNF4G_Cas_9_EV_CRI01_2.fq.gz
jc8382_HPAFII_HNF4G_Cas_9_EV_CRI02_1.fq.gz
jc8382_HPAFII_HNF4G_Cas_9_EV_CRI02_2.fq.gz
jc8384_HPAFII_H3K27Ac_Cas_9_EV_CRI01_1.fq.gz
jc8384_HPAFII_H3K27Ac_Cas_9_EV_CRI01_2.fq.gz
jc8384_HPAFII_H3K27Ac_Cas_9_EV_CRI02_1.fq.gz
jc8384_HPAFII_H3K27Ac_Cas_9_EV_CRI02_2.fq.gz
jc8385_HPAFII_H3K27Ac_Cas_9_FOXA1_CRI01_1.fq.gz
jc8385_HPAFII_H3K27Ac_Cas_9_FOXA1_CRI01_2.fq.gz
jc8385_HPAFII_H3K27Ac_Cas_9_FOXA1_CRI02_1.fq.gz
jc8385_HPAFII_H3K27Ac_Cas_9_FOXA1_CRI02_2.fq.gz
jc8385_HPAFII_H3K27Ac_Cas_9_FOXA1_CRI11_1.fq.gz
jc8385_HPAFII_H3K27Ac_Cas_9_FOXA1_CRI11_2.fq.gz
jc8385_HPAFII_H3K27Ac_Cas_9_FOXA1_CRI12_1.fq.gz
jc8385_HPAFII_H3K27Ac_Cas_9_FOXA1_CRI12_2.fq.gz
jc8386_HPAFII_H3K27Ac_HNF4G_KO_FOXA1_CRI01_1.fq.gz
jc8386_HPAFII_H3K27Ac_HNF4G_KO_FOXA1_CRI01_2.fq.gz
jc8386_HPAFII_H3K27Ac_HNF4G_KO_FOXA1_CRI02_1.fq.gz
jc8386_HPAFII_H3K27Ac_HNF4G_KO_FOXA1_CRI02_2.fq.gz
jc8387_HPAFII_Cas_9_EV_input_CRI01_1.fq.gz
jc8387_HPAFII_Cas_9_EV_input_CRI01_2.fq.gz
jc8387_HPAFII_Cas_9_EV_input_CRI02_1.fq.gz
jc8387_HPAFII_Cas_9_EV_input_CRI02_2.fq.gz
jc8387_HPAFII_Cas_9_EV_input_CRI11_1.fq.gz
jc8387_HPAFII_Cas_9_EV_input_CRI11_2.fq.gz
jc8387_HPAFII_Cas_9_EV_input_CRI12_1.fq.gz
jc8387_HPAFII_Cas_9_EV_input_CRI12_2.fq.gz
jc8388_HPAFII_Cas_9_FOXA1_input_CRI01_1.fq.gz
jc8388_HPAFII_Cas_9_FOXA1_input_CRI01_2.fq.gz
jc8388_HPAFII_Cas_9_FOXA1_input_CRI02_1.fq.gz
jc8388_HPAFII_Cas_9_FOXA1_input_CRI02_2.fq.gz
jc8389_HPAFII_HNF4G_KO_FOXA1_input_CRI01_1.fq.gz
jc8389_HPAFII_HNF4G_KO_FOXA1_input_CRI01_2.fq.gz
jc8389_HPAFII_HNF4G_KO_FOXA1_input_CRI02_1.fq.gz
jc8389_HPAFII_HNF4G_KO_FOXA1_input_CRI02_2.fq.gz

jc8389_HPAFII_HNF4G_KO_FOXA1_input_CRI11_1.fq.gz
jc8389_HPAFII_HNF4G_KO_FOXA1_input_CRI11_2.fq.gz
jc8389_HPAFII_HNF4G_KO_FOXA1_input_CRI12_1.fq.gz
jc8389_HPAFII_HNF4G_KO_FOXA1_input_CRI12_2.fq.gz
jc8390_HPAFII_H3K27Ac_Cas_9_EV_CRI01_1.fq.gz
jc8390_HPAFII_H3K27Ac_Cas_9_EV_CRI01_2.fq.gz
jc8390_HPAFII_H3K27Ac_Cas_9_EV_CRI02_1.fq.gz
jc8390_HPAFII_H3K27Ac_Cas_9_EV_CRI02_2.fq.gz
jc8391_HPAFII_H3K27Ac_Cas_9_FOXA1_CRI01_1.fq.gz
jc8391_HPAFII_H3K27Ac_Cas_9_FOXA1_CRI01_2.fq.gz
jc8391_HPAFII_H3K27Ac_Cas_9_FOXA1_CRI02_1.fq.gz
jc8391_HPAFII_H3K27Ac_Cas_9_FOXA1_CRI02_2.fq.gz
jc8392_HPAFII_H3K27Ac_HNF4G_KO_FOXA1_CRI01_1.fq.gz
jc8392_HPAFII_H3K27Ac_HNF4G_KO_FOXA1_CRI01_2.fq.gz
jc8392_HPAFII_H3K27Ac_HNF4G_KO_FOXA1_CRI02_1.fq.gz
jc8392_HPAFII_H3K27Ac_HNF4G_KO_FOXA1_CRI02_2.fq.gz
jc8392_HPAFII_H3K27Ac_HNF4G_KO_FOXA1_CRI11_1.fq.gz
jc8392_HPAFII_H3K27Ac_HNF4G_KO_FOXA1_CRI11_2.fq.gz
jc8392_HPAFII_H3K27Ac_HNF4G_KO_FOXA1_CRI12_1.fq.gz
jc8392_HPAFII_H3K27Ac_HNF4G_KO_FOXA1_CRI12_2.fq.gz
jc8393_HPAFII_H3K27Ac_Cas_9_EV_CRI01_1.fq.gz
jc8393_HPAFII_H3K27Ac_Cas_9_EV_CRI01_2.fq.gz
jc8393_HPAFII_H3K27Ac_Cas_9_EV_CRI02_1.fq.gz
jc8393_HPAFII_H3K27Ac_Cas_9_EV_CRI02_2.fq.gz
jc8394_HPAFII_H3K27Ac_Cas_9_FOXA1_CRI01_1.fq.gz
jc8394_HPAFII_H3K27Ac_Cas_9_FOXA1_CRI01_2.fq.gz
jc8394_HPAFII_H3K27Ac_Cas_9_FOXA1_CRI02_1.fq.gz
jc8394_HPAFII_H3K27Ac_Cas_9_FOXA1_CRI02_2.fq.gz
jc8395_HPAFII_H3K27Ac_HNF4G_KO_FOXA1_CRI01_1.fq.gz
jc8395_HPAFII_H3K27Ac_HNF4G_KO_FOXA1_CRI01_2.fq.gz
jc8395_HPAFII_H3K27Ac_HNF4G_KO_FOXA1_CRI02_1.fq.gz
jc8395_HPAFII_H3K27Ac_HNF4G_KO_FOXA1_CRI02_2.fq.gz
jc8396_HPAFII_flag_Cas_9_EV_CRI01_1.fq.gz
jc8396_HPAFII_flag_Cas_9_EV_CRI01_2.fq.gz
jc8396_HPAFII_flag_Cas_9_EV_CRI02_1.fq.gz
jc8396_HPAFII_flag_Cas_9_EV_CRI02_2.fq.gz
jc8397_HPAFII_flag_Cas_9_FOXA1_CRI01_1.fq.gz
jc8397_HPAFII_flag_Cas_9_FOXA1_CRI01_2.fq.gz
jc8397_HPAFII_flag_Cas_9_FOXA1_CRI02_1.fq.gz
jc8397_HPAFII_flag_Cas_9_FOXA1_CRI02_2.fq.gz
jc8398_HPAFII_flag_HNF4G_KO_FOXA1_CRI01_1.fq.gz
jc8398_HPAFII_flag_HNF4G_KO_FOXA1_CRI01_2.fq.gz
jc8398_HPAFII_flag_HNF4G_KO_FOXA1_CRI02_1.fq.gz
jc8398_HPAFII_flag_HNF4G_KO_FOXA1_CRI02_2.fq.gz
jc8398_HPAFII_flag_HNF4G_KO_FOXA1_CRI11_1.fq.gz
jc8398_HPAFII_flag_HNF4G_KO_FOXA1_CRI11_2.fq.gz
jc8398_HPAFII_flag_HNF4G_KO_FOXA1_CRI12_1.fq.gz
jc8398_HPAFII_flag_HNF4G_KO_FOXA1_CRI12_2.fq.gz
jc8399_HPAFII_flag_Cas_9_EV_CRI01_1.fq.gz
jc8399_HPAFII_flag_Cas_9_EV_CRI01_2.fq.gz
jc8399_HPAFII_flag_Cas_9_EV_CRI02_1.fq.gz
jc8399_HPAFII_flag_Cas_9_EV_CRI02_2.fq.gz
jc8399_HPAFII_flag_Cas_9_EV_CRI11_1.fq.gz
jc8399_HPAFII_flag_Cas_9_EV_CRI11_2.fq.gz
jc8399_HPAFII_flag_Cas_9_EV_CRI12_1.fq.gz
jc8399_HPAFII_flag_Cas_9_EV_CRI12_2.fq.gz
jc8400_HPAFII_flag_Cas_9_FOXA1_CRI01_1.fq.gz
jc8400_HPAFII_flag_Cas_9_FOXA1_CRI01_2.fq.gz
jc8400_HPAFII_flag_Cas_9_FOXA1_CRI02_1.fq.gz
jc8400_HPAFII_flag_Cas_9_FOXA1_CRI02_2.fq.gz
jc8400_HPAFII_flag_Cas_9_FOXA1_CRI11_1.fq.gz
jc8400_HPAFII_flag_Cas_9_FOXA1_CRI11_2.fq.gz
jc8400_HPAFII_flag_Cas_9_FOXA1_CRI12_1.fq.gz
jc8400_HPAFII_flag_Cas_9_FOXA1_CRI12_2.fq.gz
jc8401_HPAFII_flag_HNF4G_KO_FOXA1_CRI01_1.fq.gz
jc8401_HPAFII_flag_HNF4G_KO_FOXA1_CRI01_2.fq.gz
jc8401_HPAFII_flag_HNF4G_KO_FOXA1_CRI02_1.fq.gz
jc8401_HPAFII_flag_HNF4G_KO_FOXA1_CRI02_2.fq.gz
jc8404_HPAFII_FOXA1_HNF4G_KO_4_plates_CRI01_1.fq.gz
jc8404_HPAFII_FOXA1_HNF4G_KO_4_plates_CRI01_2.fq.gz
jc8404_HPAFII_FOXA1_HNF4G_KO_4_plates_CRI02_1.fq.gz
jc8404_HPAFII_FOXA1_HNF4G_KO_4_plates_CRI02_2.fq.gz
jc8405_HPAFII_FOXA1_Cas_9_4_plates_CRI01_1.fq.gz
jc8405_HPAFII_FOXA1_Cas_9_4_plates_CRI01_2.fq.gz
jc8405_HPAFII_FOXA1_Cas_9_4_plates_CRI02_1.fq.gz
jc8405_HPAFII_FOXA1_Cas_9_4_plates_CRI02_2.fq.gz

jc8405_HPAFII_FOXA1_Cas_9_4_plates_CRI11_1.fq.gz
jc8405_HPAFII_FOXA1_Cas_9_4_plates_CRI11_2.fq.gz
jc8405_HPAFII_FOXA1_Cas_9_4_plates_CRI12_1.fq.gz
jc8405_HPAFII_FOXA1_Cas_9_4_plates_CRI12_2.fq.gz
jc8406_HPAFII_FOXA1_HNF4G_KO_4_plates_CRI01_1.fq.gz
jc8406_HPAFII_FOXA1_HNF4G_KO_4_plates_CRI01_2.fq.gz
jc8406_HPAFII_FOXA1_HNF4G_KO_4_plates_CRI02_1.fq.gz
jc8406_HPAFII_FOXA1_HNF4G_KO_4_plates_CRI02_2.fq.gz
jc8406_HPAFII_FOXA1_HNF4G_KO_4_plates_CRI11_1.fq.gz
jc8406_HPAFII_FOXA1_HNF4G_KO_4_plates_CRI11_2.fq.gz
jc8406_HPAFII_FOXA1_HNF4G_KO_4_plates_CRI12_1.fq.gz
jc8406_HPAFII_FOXA1_HNF4G_KO_4_plates_CRI12_2.fq.gz
jc8407_HPAFII_FOXA1_Cas_9_2_plates_CRI01_1.fq.gz
jc8407_HPAFII_FOXA1_Cas_9_2_plates_CRI01_2.fq.gz
jc8407_HPAFII_FOXA1_Cas_9_2_plates_CRI02_1.fq.gz
jc8407_HPAFII_FOXA1_Cas_9_2_plates_CRI02_2.fq.gz
jc8407_HPAFII_FOXA1_Cas_9_2_plates_CRI11_1.fq.gz
jc8407_HPAFII_FOXA1_Cas_9_2_plates_CRI11_2.fq.gz
jc8407_HPAFII_FOXA1_Cas_9_2_plates_CRI12_1.fq.gz
jc8407_HPAFII_FOXA1_Cas_9_2_plates_CRI12_2.fq.gz
jc8408_HPAFII_FOXA1_HNF4G_KO_2_plates_CRI01_1.fq.gz
jc8408_HPAFII_FOXA1_HNF4G_KO_2_plates_CRI01_2.fq.gz
jc8408_HPAFII_FOXA1_HNF4G_KO_2_plates_CRI02_1.fq.gz
jc8408_HPAFII_FOXA1_HNF4G_KO_2_plates_CRI02_2.fq.gz
jc8409_HPAFII_FOXA1_Cas_9_4_plates_CRI01_1.fq.gz
jc8409_HPAFII_FOXA1_Cas_9_4_plates_CRI01_2.fq.gz
jc8409_HPAFII_FOXA1_Cas_9_4_plates_CRI02_1.fq.gz
jc8409_HPAFII_FOXA1_Cas_9_4_plates_CRI02_2.fq.gz
jc8412_HPAFII_HNF4G_HNF4G_KO_4_plates_CRI01_1.fq.gz
jc8412_HPAFII_HNF4G_HNF4G_KO_4_plates_CRI01_2.fq.gz
jc8412_HPAFII_HNF4G_HNF4G_KO_4_plates_CRI02_1.fq.gz
jc8412_HPAFII_HNF4G_HNF4G_KO_4_plates_CRI02_2.fq.gz
jc8414_HPAFII_HNF4G_HNF4G_KO_4_plates_CRI01_1.fq.gz
jc8414_HPAFII_HNF4G_HNF4G_KO_4_plates_CRI01_2.fq.gz
jc8414_HPAFII_HNF4G_HNF4G_KO_4_plates_CRI02_1.fq.gz
jc8414_HPAFII_HNF4G_HNF4G_KO_4_plates_CRI02_2.fq.gz
jc8415_HPAFII_Cas_9_input_CRI01_1.fq.gz
jc8415_HPAFII_Cas_9_input_CRI01_2.fq.gz
jc8415_HPAFII_Cas_9_input_CRI02_1.fq.gz
jc8415_HPAFII_Cas_9_input_CRI02_2.fq.gz
jc8416_HPAFII_HNF4G_KO_input_CRI01_1.fq.gz
jc8416_HPAFII_HNF4G_KO_input_CRI01_2.fq.gz
jc8416_HPAFII_HNF4G_KO_input_CRI02_1.fq.gz
jc8416_HPAFII_HNF4G_KO_input_CRI02_2.fq.gz

Genome browser session (e.g. UCSC)

hg38,mm10

Methodology

Replicates

A minimum of three biological replicates were performed for DiffBind analyses.

Sequencing depth

NovaSeq 6000 was used for the ChIP-seq experiments. 20-30 million reads were aimed for most samples incorporated into the study.

Antibodies

HNF4G (Atlas, HPA005438), HNF4A(Cell signalling, 3113S), PRMT1(Atlas, HPA072136), GATA6 (Cell signalling 5851, R&D systems AF1700) FOXA1 (abcam, ab5089)

Peak calling parameters

macs2 callpeak -t <chip> -c <input> -f BAM -g hs -n <chip name>-X-<input name> -q 0.05 -m 5 50 --nomodel

Data quality

Peak quality was assessed with quality evaluation tool which is the integral part of analysis pipeline.

Software

MACS2 v 2.2.6, bedtools v 2.26.0-97, DiffBind v 2.2.12, Meme 4.9.1,5.0.5

Flow Cytometry

Plots

Confirm that:

☐ The axis labels state the marker and fluorochrome used (e.g. CD4-FITC).

☐ The axis scales are clearly visible. Include numbers along axes only for bottom left plot of group (a 'group' is an analysis of identical markers).

☐ All plots are contour plots with outliers or pseudocolor plots.

☐ A numerical value for number of cells or percentage (with statistics) is provided.

Methodology

Sample preparation	*Describe the sample preparation, detailing the biological source of the cells and any tissue processing steps used.*
Instrument	*Identify the instrument used for data collection, specifying make and model number.*
Software	*Describe the software used to collect and analyze the flow cytometry data. For custom code that has been deposited into a community repository, provide accession details.*
Cell population abundance	*Describe the abundance of the relevant cell populations within post-sort fractions, providing details on the purity of the samples and how it was determined.*
Gating strategy	*Describe the gating strategy used for all relevant experiments, specifying the preliminary FSC/SSC gates of the starting cell population, indicating where boundaries between "positive" and "negative" staining cell populations are defined.*

☐ Tick this box to confirm that a figure exemplifying the gating strategy is provided in the Supplementary Information.

Magnetic resonance imaging

Experimental design

Design type	*Indicate task or resting state; event-related or block design.*
Design specifications	*Specify the number of blocks, trials or experimental units per session and/or subject, and specify the length of each trial or block (if trials are blocked) and interval between trials.*
Behavioral performance measures	*State number and/or type of variables recorded (e.g. correct button press, response time) and what statistics were used to establish that the subjects were performing the task as expected (e.g. mean, range, and/or standard deviation across subjects).*

Acquisition

Imaging type(s)	*Specify: functional, structural, diffusion, perfusion.*
Field strength	*Specify in Tesla*
Sequence & imaging parameters	*Specify the pulse sequence type (gradient echo, spin echo, etc.), imaging type (EPI, spiral, etc.), field of view, matrix size, slice thickness, orientation and TE/TR/flip angle.*
Area of acquisition	*State whether a whole brain scan was used OR define the area of acquisition, describing how the region was determined.*

Diffusion MRI ☐ Used ☐ Not used

Preprocessing

Preprocessing software	*Provide detail on software version and revision number and on specific parameters (model/functions, brain extraction, segmentation, smoothing kernel size, etc.).*
Normalization	*If data were normalized/standardized, describe the approach(es): specify linear or non-linear and define image types used for transformation OR indicate that data were not normalized and explain rationale for lack of normalization.*
Normalization template	*Describe the template used for normalization/transformation, specifying subject space or group standardized space (e.g. original Talairach, MNI305, ICBM152) OR indicate that the data were not normalized.*
Noise and artifact removal	*Describe your procedure(s) for artifact and structured noise removal, specifying motion parameters, tissue signals and physiological signals (heart rate, respiration).*

Volume censoring | *Define your software and/or method and criteria for volume censoring, and state the extent of such censoring.*

Statistical modeling & inference

Model type and settings | *Specify type (mass univariate, multivariate, RSA, predictive, etc.) and describe essential details of the model at the first and second levels (e.g. fixed, random or mixed effects; drift or auto-correlation).*

Effect(s) tested | *Define precise effect in terms of the task or stimulus conditions instead of psychological concepts and indicate whether ANOVA or factorial designs were used.*

Specify type of analysis: ☐ Whole brain ☐ ROI-based ☐ Both

Statistic type for inference | *Specify voxel-wise or cluster-wise and report all relevant parameters for cluster-wise methods.*

(See Eklund et al. 2016)

Correction | *Describe the type of correction and how it is obtained for multiple comparisons (e.g. FWE, FDR, permutation or Monte Carlo).*

Models & analysis

n/a	Involved in the study
☒	☐ Functional and/or effective connectivity
☒	☐ Graph analysis
☒	☐ Multivariate modeling or predictive analysis
