# Supplementary material for: Transcription factor switching drives subtype-specific pancreatic cancer
Source: Nat Genet. 2025 Oct 30;57(12):3016–26. doi: 10.1038/s41588-025-02389-7 (PMC12695649; doi:10.1038/s41588-025-02389-7)

Extended Supplementary Figure 2d

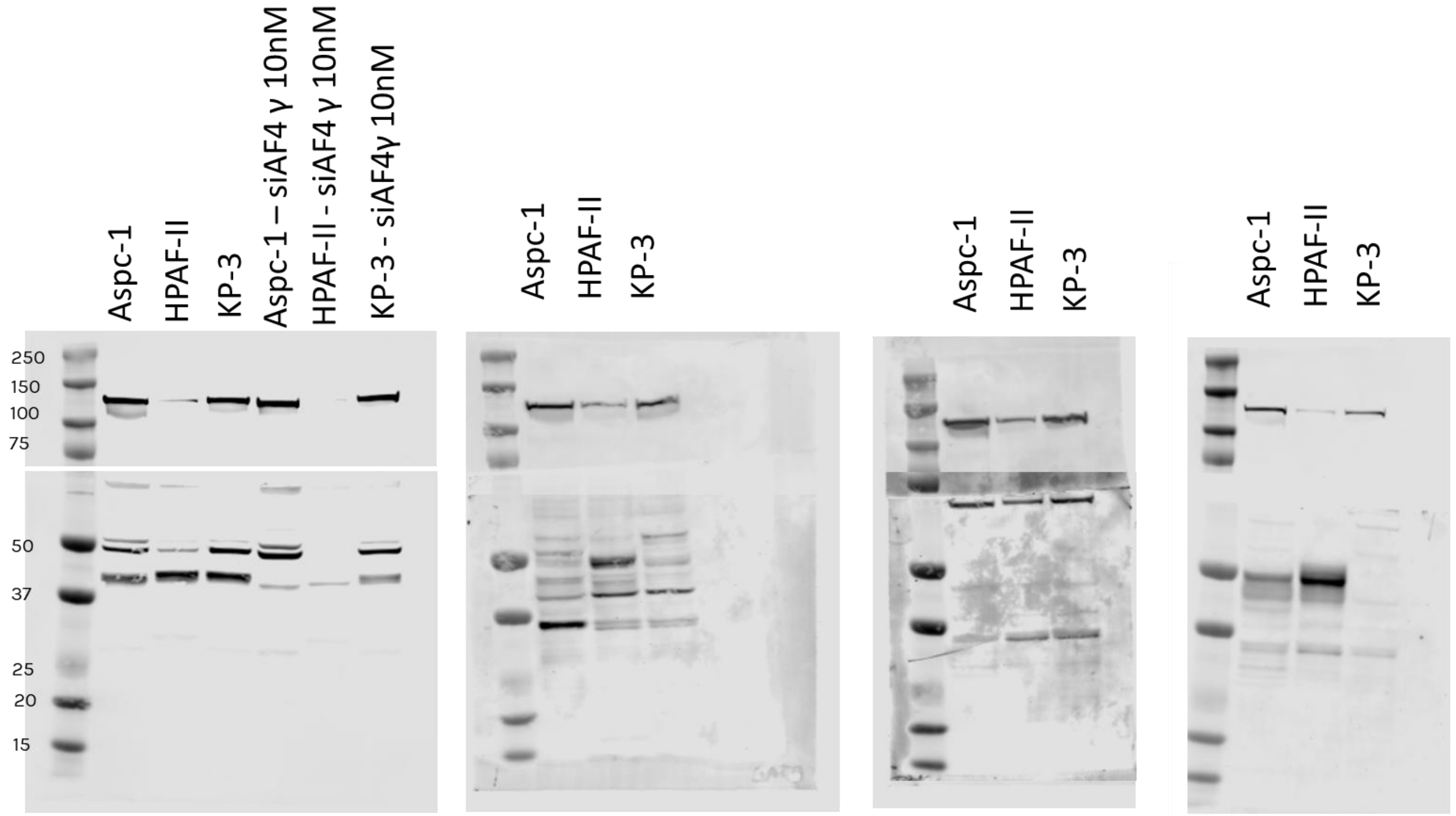

Vinculin (124kDa)
Ab0029.2
Cell Signalling Technology
Rabbit 1:2000

HNF4G (46 kDa)
Ab0118
Proteintech
Rabbit 1:600

FOXA1 (50 kDa)
ab23738
Abcam
Rabbit 1:1000

GATA6 (55 kDa)
ab22600
Abcam
Rabbit

HNF4A (52 kDa)
Ab0121
Cell Signalling Technology
Rabbit 1:1000

20μg total protein loaded per lane (10well, 1.0mm, 4-12% Bis/Tris gel), 1x MOPS running buffer, Semi-wet transfer

Extended Supplementary Figure 2i

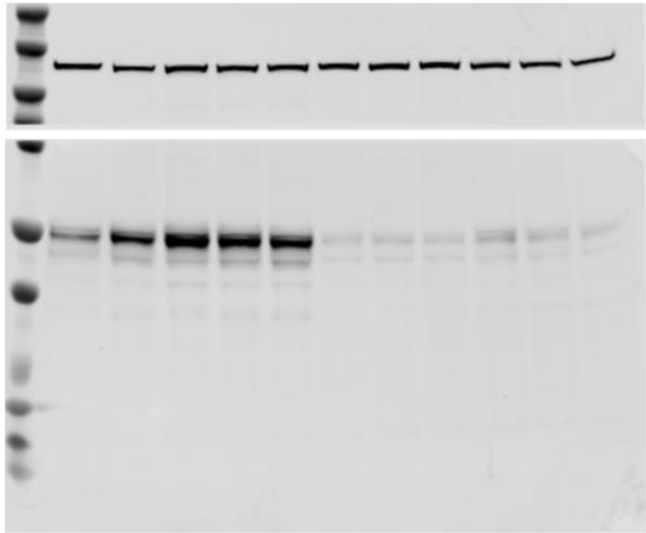

←Vinculin

Vinculin (124kDa)
Cell Signaling Technology
Rabbit 1:2000

←HNF4A

AF4 α (52 kDa)
Cell Signalling Technology
Rabbit 1:1000

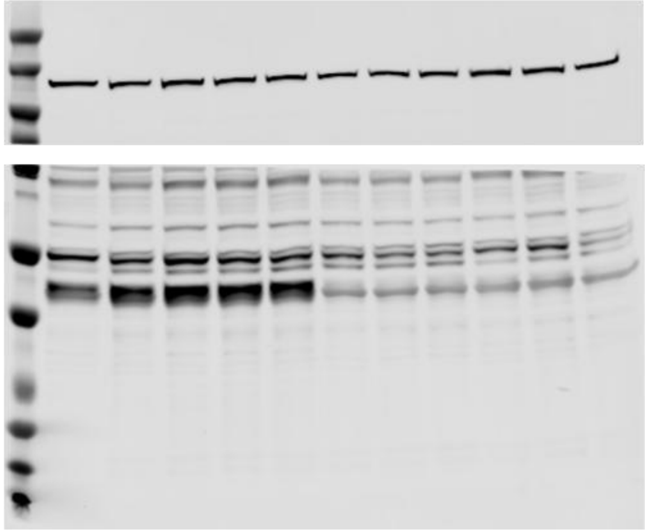

←Vinculin

Vinculin (124kDa)
Cell Signaling Technology
Rabbit 1:2000

←HNF4G

HNF4G (46 kDa)
Proteintech
Rabbit 1:600

Extended Supplementary Figure 3e

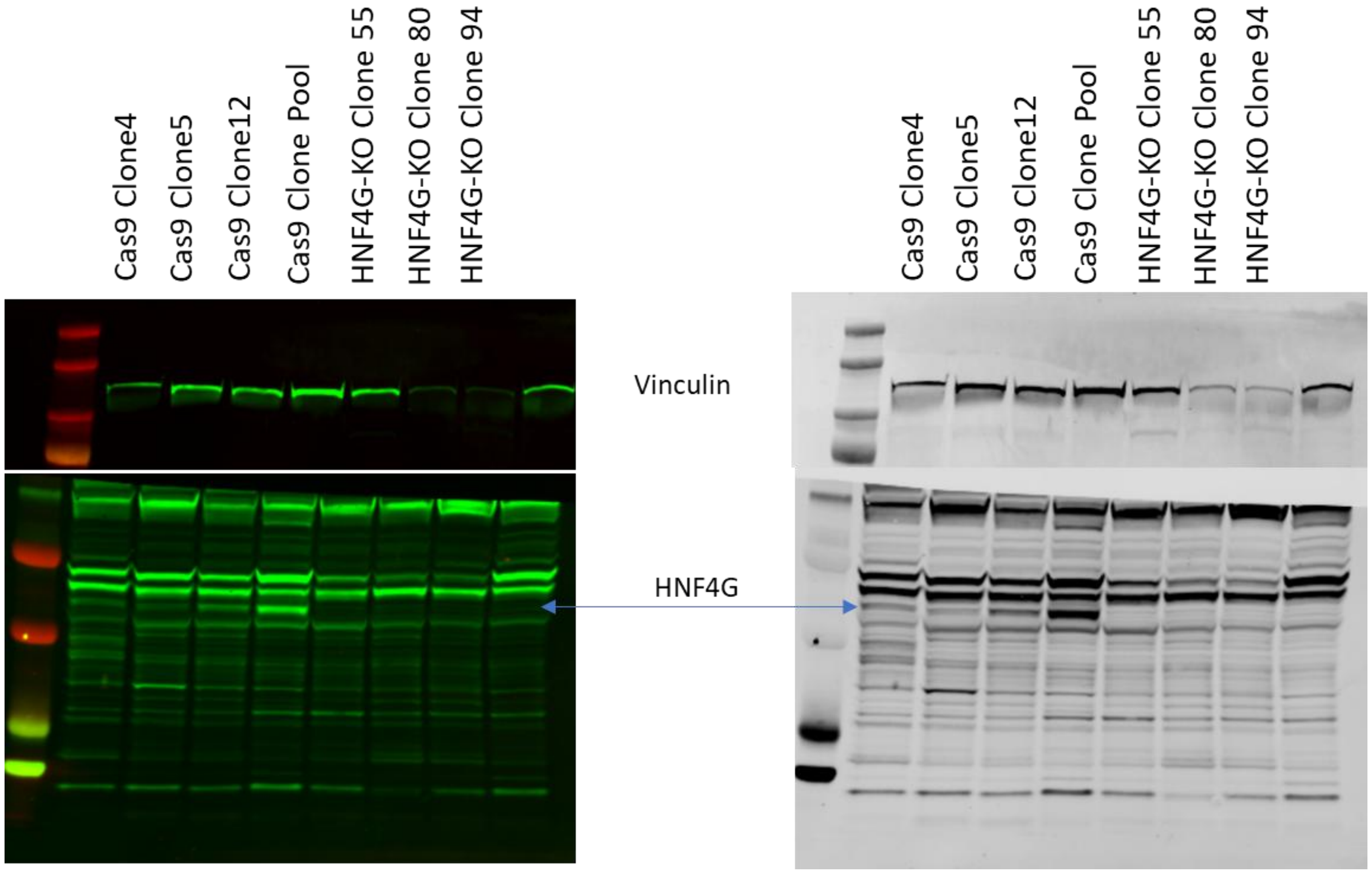

Extended Supplementary Figure 4b

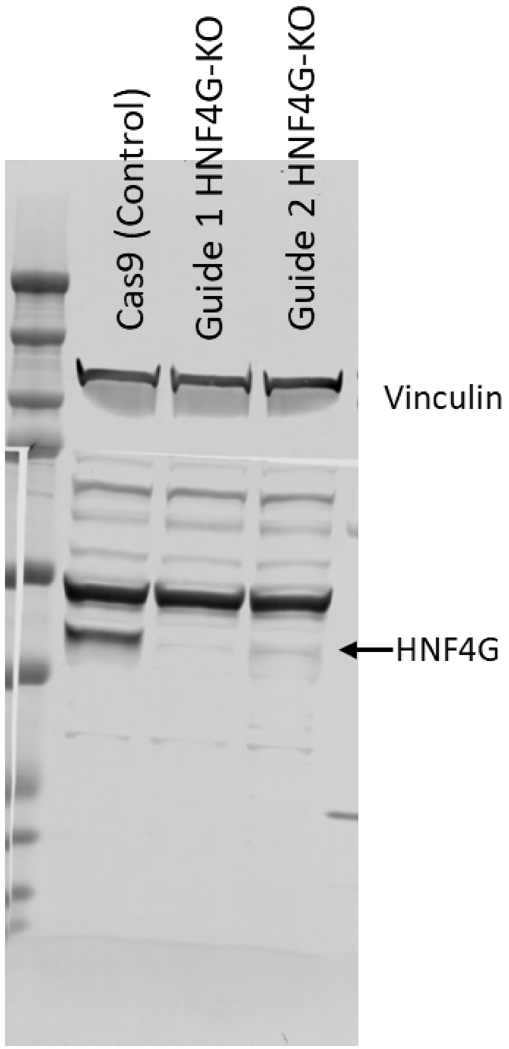

Extended Supplementary Figure 4f

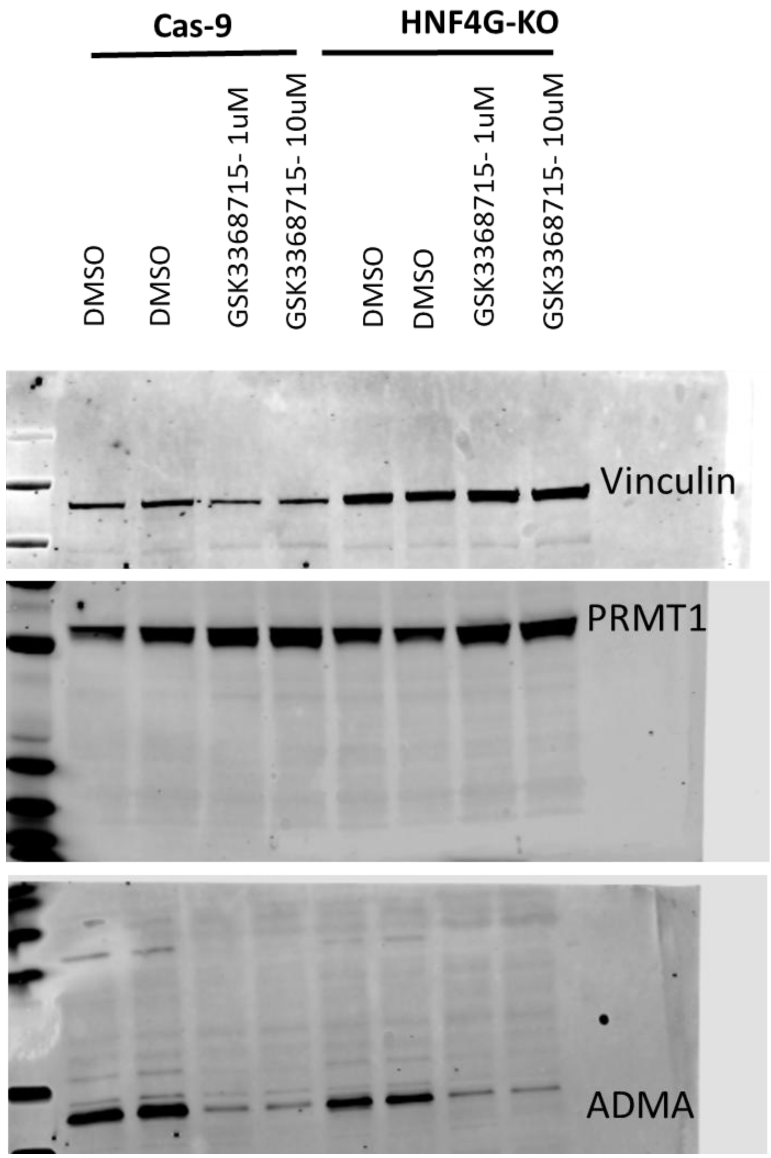

Supplement: Supplementary file 3 — Source data for Extended Data Figs. 2d,i, 3e and 4b,f. [file 41588_2025_2389_MOESM3_ESM.pdf]
